# Paleopolyploidies and Genomic Fractionation in Major Eudicot Clades

**DOI:** 10.3389/fpls.2022.883140

**Published:** 2022-05-31

**Authors:** Jia Teng, Jianyu Wang, Lan Zhang, Chendan Wei, Shaoqi Shen, Qimeng Xiao, Yuanshuai Yue, Yanan Hao, Weina Ge, Jinpeng Wang

**Affiliations:** ^1^Department of Bioinformatics, School of Life Sciences and Center for Genomics and Computational Biology, North China University of Science and Technology, Tangshan, China; ^2^State Key Laboratory of Systematic and Evolutionary Botany, Institute of Botany, Chinese Academy of Science, Beijing, China

**Keywords:** *Aquilegia coerulea*, *Nelumbo nucifera*, *Vitis vinifera*, hierarchical alignment, gene colinearity, polyploidization

## Abstract

Eudicots account for ~75% of living angiosperms, containing important food and energy crops. Recently, high-quality genome sequences of several eudicots including *Aquilegia coerulea* and *Nelumbo nucifera* have become available, providing an opportunity to investigate the early evolutionary characteristics of eudicots. We performed genomic hierarchical and event-related alignments to infer homology within and between representative species of eudicots. The results provide strong evidence for multiple independent polyploidization events during the early diversification of eudicots, three of which are likely to be allopolyploids: The core eudicot-common hexaploidy (ECH), *Nelumbo*-specific tetraploidy (NST), and Ranunculales-common tetraploidy (RCT). Using different genomes as references, we constructed genomic alignment to list the orthologous and paralogous genes produced by polyploidization and speciation. This could provide a fundamental framework for studying other eudicot genomes and gene(s) evolution. Further, we revealed significantly divergent evolutionary rates among these species. By performing evolutionary rate correction, we dated RCT to be ~118–134 million years ago (Mya), after Ranunculales diverged with core eudicots at ~123–139 Mya. Moreover, we characterized genomic fractionation resulting from gene loss and retention after polyploidizations. Notably, we revealed a high degree of divergence between subgenomes. In particular, synonymous nucleotide substitutions at synonymous sites (*Ks*) and phylogenomic analyses implied that *A. coerulea* might provide the subgenome(s) for the *gamma*-hexaploid hybridization.

## Introduction

There are around 280,000 species of eudicots, accounting for ~75% of all living angiosperms (Zeng et al., 2014). Ranunculales, Proteales, Trochodendrales, and Buxales are four sister groups of core eudicots (Bremer et al., 2009; Byng et al., 2016). Among all sequenced core eudicots, *Vitis vinifera* (2n = 38) was the first sequenced fruit crop. There are relatively few structural changes in its genome (Jaillon et al., 2007), and it is often selected as a good reference for research on the evolution of eudicots. Recently, several high-quality whole genomes from basal eudicots have been sequenced or updated. *Nelumbo nucifera* (2n = 16) is a member of the family Nelumbonaceae from order Proteales, which was first sequenced in 2013 (Ming et al., 2013) and has further been updated in assembly (Gui et al., 2018; Shi et al., 2020). *Macadamia integrifolia* (2n = 28) is a representative species of Proteales and its genome has been sequenced (Nock et al., 2020). Ranunculales is the earliest group diverging from other eudicots, including the genomes of *Aquilegia coerulea* (2n = 14) (Aköz and Nordborg, 2019), *Aquilegia oxysepala* var. *kansuensis* (2n = 14) (Xie et al., 2020), and *Papaver somniferum* L. (2n = 22) (Guo et al., 2018) which are already available. *Tetracentron sinense* (2n = 48) is a member of Trochodendrales, an endemic and endangered deciduous tree with the genome sequenced (Liu et al., 2020). All these genomes are chromosome-level assembly and located at the critical phylogenetic positions of eudicots, facilitating to elucidate key evolutionary features during the early evolution of eudicots.

Polyploidy, or whole-genome duplication (WGD), is a key driver in species evolution and occurs widely in angiosperms (Paterson et al., 2004; Jiao et al., 2011; Jiao and Paterson, 2014; Murat et al., 2017). Previous studies have revealed that an ancient hexaploidy event, known as *gamma* (Vision et al., 2000; Bowers et al., 2003; Jaillon et al., 2007; Tang et al., 2008), occurred within a few million years during the early diversification of eudicots (Bell et al., 2010; Magallon et al., 2015). As yet, among all identified WGDs, the *gamma* event might affect the largest number of eudicots' plant groups (Jiao et al., 2011). After WGD, there are complex chromosomal rearrangements (Murat et al., 2017), a large number of duplicated genes are lost, and sequence divergence occurs. These are important processes in promoting genetic innovation (Puchta et al., 1996; Long et al., 2003; Mitchell-Olds and Schmitt, 2006), but make genomes extremely complex (Wang et al., 2017, 2018b, 2019b, 2020a). This may lead to problematic interpretations of the evolution of WGD events (Wang et al., 2005, 2016; Li et al., 2015). Early divergence of eudicots occurred at ~130**–**150 Mya (Jiao et al., 2011; Magallon et al., 2015). Therefore, the major eudicot clades diverged in a relatively short period compared to their entire evolutionary history, which also constitutes a challenge for deciphering the *gamma* event.

Several studies have sought to narrow the phylogenetic placement of the *gamma* event, and it was determined to be shared by the core eudicots (Bowers et al., 2003; Jaillon et al., 2007; Lyons et al., 2008; Jiao et al., 2012; Ming et al., 2013). In contrast to this, a recent study reported that *A. coerulea* share an ancient tetraploid with core eudicots, and this tetraploid was hybridized with a diploid to form the *gamma* hexaploid (Aköz and Nordborg, 2019). However, another report re-identified the placement of the *Aquilegia* WGD and found that it is more likely to be lineage-specific (Shi and Chen, 2020). Besides, the ancient WGD of *P. somniferum* is still unclear. *P. somniferum* (high noscapine 1, HN1) genome sequencing and analyses suggested an ancient segmental or whole-genome duplication(s) that occurred in *P. somniferum* before the Papaveraceae-Ranunculaceae divergence (Guo et al., 2018). Another variety of *P. somniferum* (Chinese Herbal Medicine, CHM) genome sequencing project implied that Ranunculales may share one common WGD (Pei et al., 2021). Focusing on *P. somniferum* WGD, Shi and Chen used phylogenomic analyses of duplicated genes and proposed that the syntenic ratio is 2:2 between *Aquilegia* and *Papaver* (Shi and Chen, 2020), supporting the independent WGD of *P. somniferum*, but not shared by *Aquilegia* and *Papaver*.

Here, representative genomes from major clades of eudicots were selected for genomic hierarchical and event-related analyses. The study aims to clarify the early evolutionary history of eudicots, examine whether there is a common WGD after eudicots divergence from monocots, investigate the divergent evolutionary rates among the species under consideration, construct the multi-genomic homology information related to polyploidization and speciation, and explore the polyploidy nature of Ranunculales-common tetraploidy (RCT), *Nelumbo*-specific tetraploidy (NST), and core eudicot-common hexaploidy (ECH).

## Results

### Colinear Genes and Characterized *Ks* Distribution

According to the latest assembled genomic version, we found that the *N. nucifera* genome has better intragenomic homology than *V. vinifera* and *A. coerulea*, and it retains more colinear genes generated by ancestral WGD (Supplementary Tables 1, 2). For homologous blocks with at least four anchor gene pairs, *N. nucifera* has 392 blocks containing 6,471 colinear gene pairs. Under the same criteria, *V. vinifera* and *A. coerulea* have relatively few colinear genes. The intergenomic collinearity is better than intragenomic, and the intergenomic homologous regions between *N. nucifera* and *V. vinifera* were better preserved than between *N. nucifera* and *A. coerulea* or *A. coerulea* and *V. vinifera* (Supplementary Tables 1, 2). For homologous blocks with four or more olinear genes, we detected 994–1,352 regions between any two of these three eudicots. The least is between *V. vinifera* and *A. coerulea*, and the most is between *N. nucifera* and *V. vinifera*.

**Figure 1:**
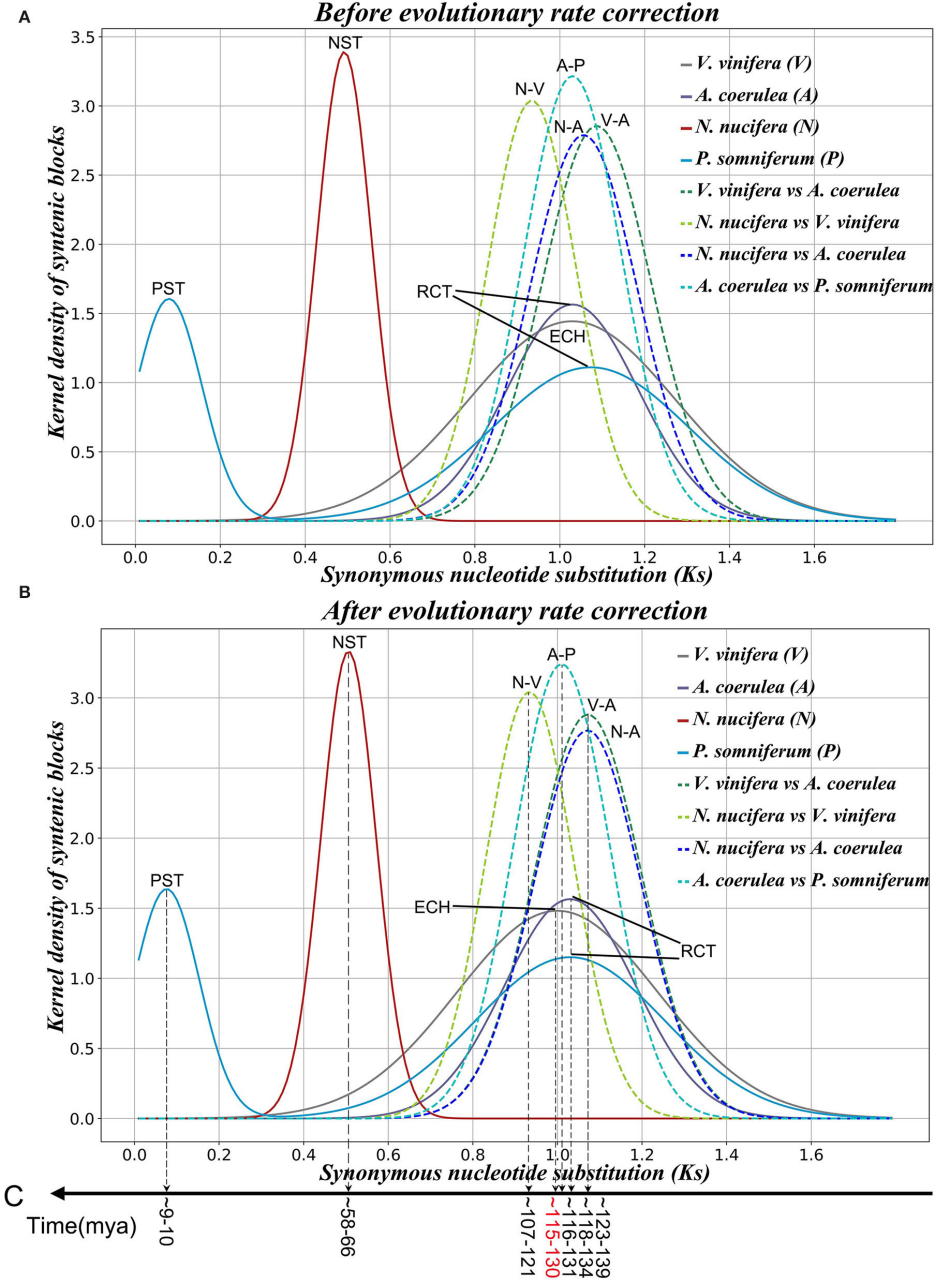
*Ks* distribution of colinear genes within and among genomes. **(A)** Before evolutionary rate correction. **(B)** After evolutionary rate correction. **(C)** Inferred ages of key evolutionary events.

Based on the *Ks* divergence between each colinear gene pair within and among genomes, we found that the peak of the *Ks* distribution between *N. nucifera* duplicated gene pairs is at ~0.492 (Supplementary Table 3). The *Ks* peak in *N. nucifera* genome (0.492) is much smaller than in *A. coerulea* (1.030) and *V. vinifera* (1.029) (Figure 1A and Supplementary Figures 1A–C), indicating that NST is apparently younger than RCT and ECH. Previous studies have reported that NST occurred ~65 Mya (Ming et al., 2013; Gui et al., 2018; Shi et al., 2020). *Ks* peaks generated by the RCT are at divergent locations in *A. coerulea* and *P. somniferum* genomes, and the ancient *Ks* peak in *P. somniferum* is slightly larger than in *A. coerulea* (Figure 1A). This suggests that *P. somniferum* evolved faster than *A. coerulea*. The younger *Ks* peak of *P. somniferum* is at ~0.079, indicating *Papaver*-specific tetraploidy (PST) in addition to RCT.

### Absence of ECH Event in *Nelumbo, Macadamia*, and *Tetracentron* Lineages

**Figure 2:**
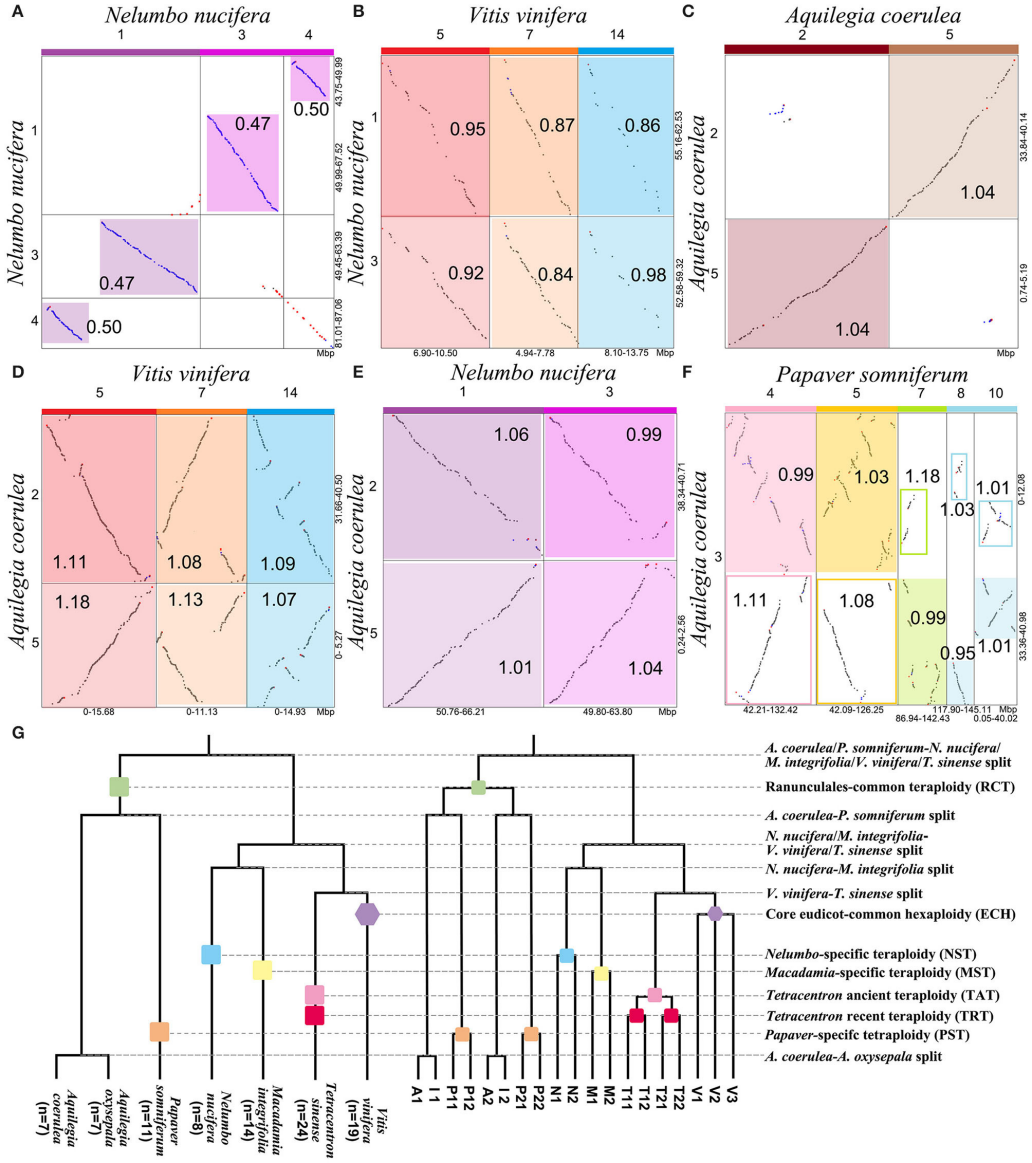
Intragenomic and intergenomic local dot plots and phylogenetic tree. Local dot plot and *Ks* medians **(A)** within *N. nucifera*, **(B)** between *V. vinifera* and *N. nucifera*, **(C)** within *A. coerulea*, **(D)** between *V. vinifera* and *A. coerulea*, **(E)** between *N. nucifera* and *A. coerulea*, and **(F)** between *A. coerulea* and *P. somniferum*. On the top of the local dot plots, the colored blocks represent the paralogous chromosomes produced by WGD. The highlighted areas of different brightness represent homologous regions, and the outparalogous regions between genomes are framed by solid lines. **(G)** Species and gene phylogenetic trees of *A. coerulea* (A), *A. oxysepala* (I), *N. nucifera* (N), *P. somniferum* (P), *M. integrifolia* (M), *T. sinense* (T), and *V. vinifera* (V). The phylogenetic relationship was based on the current accepted topology (Angiosperm Phylogeny Website). A number of three paralogous genes produced by the ECH in the *V. vinifera* genome are denoted by V1, V2, and V3, and each has two orthologous genes in *N. nucifera, A. coerulea*, and *M. integrifolia*, and four in *P. somniferum* and *T. sinense*.

We constructed an intragenomic dot plot of *N. nucifera* and an intergenomic dot plot of *N. nucifera* and *V. vinifera* genomes, showing large homologous chromosomal blocks with the *Ks* medians of colinear gene pairs. The *Ks* medians of paralogous regions in *N. nucifera* genome are around 0.492, corresponding to the NST event (Figure 1A, Supplementary Figure 3, and Supplementary Table 3). The genomic colinear depth ratio is 1:1 within *N. nucifera* genome, that is, a *N. nucifera* gene (or chromosome region) corresponds to only one best matched homologous gene (or chromosome region) within the genome; just like the local dot plot show the region of *N. nucifera* chromosome 1 (49.99–67.52 Mb) corresponds to one best region is chromosome 3 (49.45–63.39 Mb) (Figure 2A). These homologous regions were generated by the more recent tetraploidy event in the ancestral genome of *N. nucifera*. Furthermore, comparative analysis of *N. nucifera*-*V. vinifera* dot plot identified the orthologous regions with *Ks* medians around 0.934, corresponding to the divergence of *N. nucifera* and *V. vinifera* (Figure 1A, Supplementary Figure 4, and Supplementary Table 3). The genomic colinear depth ratio of *N. nucifera*-*V. vinifera* is 2:3, that is, a pair of paralogous genes (or chromosomal regions) generated by NST in *N. nucifera* genome corresponds to three best matched genes (or orthologous regions) in *V. vinifera* (Figure 2B). The genomic colinear ratio confirms that there is no hexaploidization in *N. nucifera*, but a lineage-specific tetraploid event occurred at ~65 Mya (Jaillon et al., 2007; Jiao et al., 2011; Ming et al., 2013).

When comparing *N. nucifera* and *M. integrifolia* genomes, we identified the orthologous regions related to the species divergence with *Ks* medians around 0.763 (Supplementary Figure 5). The colinear depth ratio of 2:2 was inferred between their genomes, indicating that a tetraploidy event occurred in *M. integrifolia* after diverged with the *N. nucifera*, that is, *Macadamia*-specific tetraploidy (MST). Besides, we identified the orthologous regions generated from the divergence of *V. vinifera* and *T. sinense* (Supplementary Figure 6). The colinear depth ratio between *V. vinifera* and *T. sinense* is 4:3, confirming that there were two *T. sinense* lineage-specific WGDs, namely, *T. sinense*-ancient tetraploidy (TAT) and *T. sinense*-recent tetraploidy (TRT), which is consistent with previous study (Liu et al., 2020). These comparisons strongly support that the *Nelumbo, Macadamia*, and *Tetracentron* lineages lack the paleohexaploidy (ECH) and the respective WGD events.

### Ranunculales-Common Tetraploidization

Through the intragenomic structure comparison of *A. coerulea*, we found longer paralogous regions with *Ks* medians around 1.030, and the best intragenomic homologous match is 1:1 (Figure 1A, Supplementary Figure 7, and Supplementary Table 3). It confirms that *A. coerulea* underwent one tetraploidization event; for example, the best match of chromosome 2 is chromosome 5, and they are paralogous regions produced by RCT (Figure 2C). In the *A. coerulea*–*V. vinifera* dot plot, the orthologous regions with *Ks* medians are around 1.087, corresponding to the divergence of them (Figure 1A, Supplementary Figure 8, and Supplementary Table 3). The colinear depth ratio between the *A. coerulea* and *V. vinifera* genomes is 2:3, that is, a pair of paralogous genes (or chromosomal regions) generated by RCT in *A. coerulea*, corresponding to three best matched genes (or orthologous regions) in *V. vinifera*, just like Figure 2D shows. The result implies that an *A. coerulea* ancestral tetraploidization event occurred after splitting with *V. vinifera*, and the ECH only covers *V. vinifera* (core eudicots).

Furthermore, strong evidence was obtained by intergenomic comparison of *A. coerulea* and *N. nucifera*. The *Ks* medians between homologous regions are about 1.056 (Supplementary Figure 9 and Supplementary Table 3), corresponding to the divergence of *A. coerulea* and *N. nucifera*, and the colinear depth ratio between them is 2:2. For example, a pair of paralogous regions in *A. coerulea* chromosomes 2 and 5 correspond to two orthologous regions in *N. nucifera* chromosomes 1 and 3 (Figure 2E). The *Ks* peak of *N. nucifera* WGD is 0.492, this is, significantly younger than *A. coerulea* WGD (1.030) (Figure 1A and Supplementary Table 3). It strongly suggests that there is no shared WGD of *N. nucifera* and *A. coerulea* after the formation of eudicots, unlike previously reported that the WGD of *A. coerulea* is shared by all eudicots (Aköz and Nordborg, 2019). If the WGD of *A. coerulea* is shared by *N. nucifera*, the colinear depth ratio between them would be 2:4, conflicting with the *Nelumbo, Macadamia*, and *Tetracentron* lineages lack shared WGD events with *A. coerulea*.

Ranunculales shared an ancient tetraploidization. The *Ks* medians of homologous regions between *A. coerulea* and *P. somniferum* genomes are around 1.030, and the colinear depth ratio between them is 2:4 (Supplementary Figure 10). Comparing *Ks* values of homologous regions, we found a pair of paralogous genes (or chromosomal regions) generated by RCT in the *A. coerulea* genome correspond to the two best matched genes (or orthologous regions) in *P. somniferum* and two secondary genes (or outparalogous regions), just like the local dot plot shows (Figures 2F,G). The orthologous syntenic depth ratio supports a further WGD event of *P. somniferum* after RCT. The older is shared by *A. coerulea* and *P. somniferum* and is likely to be RCT, whereas the younger WGD occurred only in *P. somniferum*. As shown in *Ks* distribution, the *Ks* peaks of *A. coerulea* WGD and *P. somniferum* older WGD are close to the peak of *A. coerulea*–*P. somniferum* divergence. This suggests that they may have immediately diverged after the shared WGD, thus rendering it very difficult to time the WGD.

### Phylogenomic Analysis

**Figure 3:**
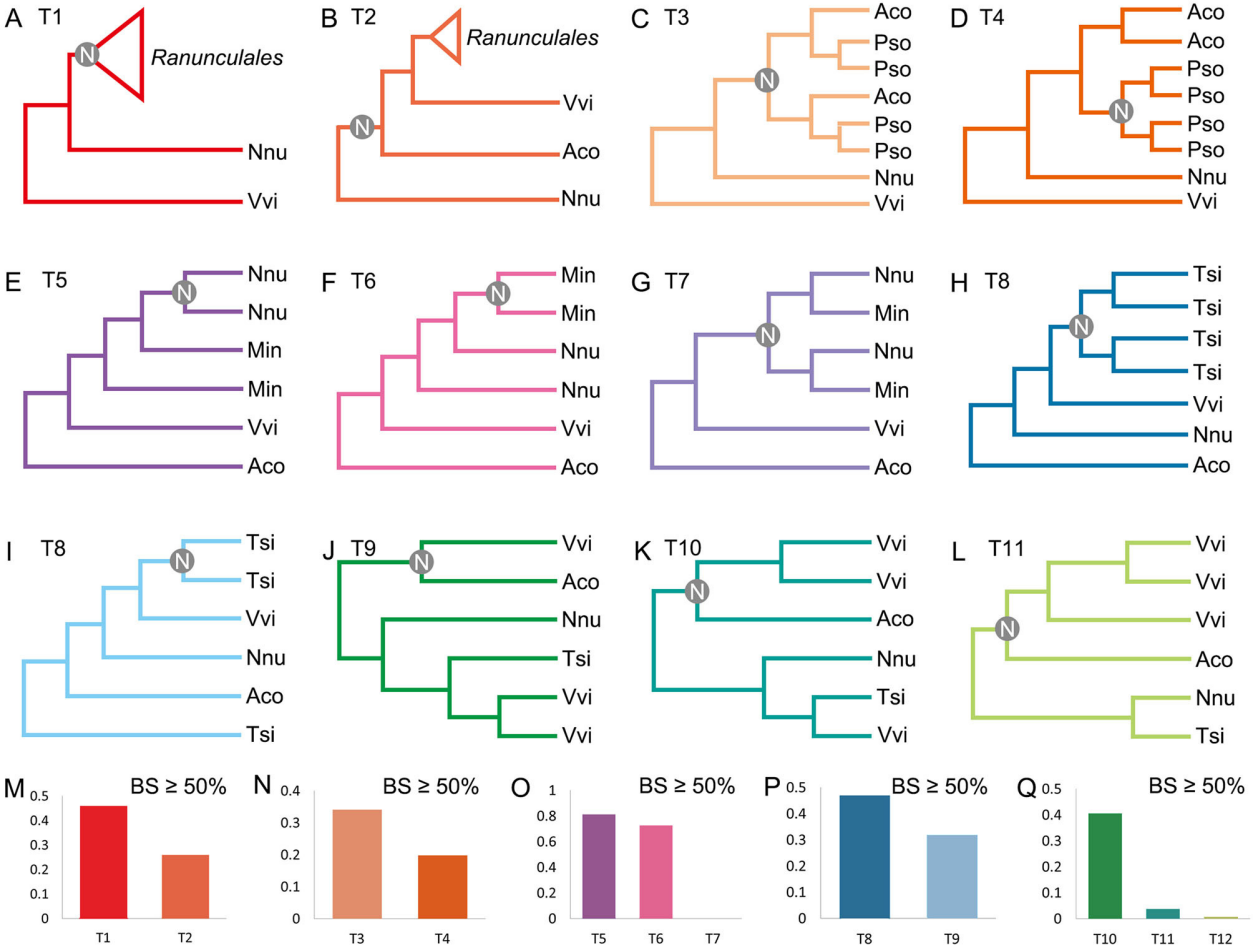
Possible topologies of gene trees and statistics of phylogenomic analysis. Topology support **(A)** WGDs of Ranunculales independently of other species, **(B)** a WGD shared by *A. coerulea* and *V. vinifera*, **(C)** RCT, **(D)** respective WGDs of *A. coerulea* and *P. somniferum*, **(E)** NST, **(F)** MST, **(G)** common WGD of *N. nucifera* and *M. integrifolia*, **(H)** two WGDs of *T. sinense*, **(I)** only one WGD of *T. sinense*, **(J)** *A. coerulea* provide one paralogous gene pair to *V. vinifera*, **(K)** *A. coerulea* provide two paralogous gene pairs to *V. vinifera*, **(L)** *A. coerulea* provide three paralogous gene pairs to *V. vinifera*. The key nodes are indicated by gray circles. **(M–Q)** Gene tree frequencies for different topologies, and BS of the key node is more than 50%. *Ranunculales* represents genes from species of Ranunculales, and Aco, Pso, Vvi, Nnu, Min, and Tsi represent genes from the genomes of *A. coerulea, P. somniferum, V. vinifera, N. nucifera, M. integrifolia*, and *T. sinense*, respectively. The *x*-axis labels T1 to T12 refer to the support for the topologies.

Phylogenetic analysis is used to further validate the polyploidizations. A constructed gene tree is considered to support a topology when the bootstrap support (BS) of the key node in the gene tree ≥50% (Figure 3). When investigating the RCT event, we constructed trees for 198 orthogroups, and each group had one pair of *A. coerulea* paralogous genes, at least three *P. somniferum*, one *V. vinifera*, and one *N. nucifera* orthologous genes to these *A. coerulea* genes. We detected that 45.96% (91/198) trees supporting the RCT occurred after the divergence of *A. coerulea* from *N. nucifera* and *V. vinifera*, whereas only 25.76% (51/198) trees supporting the RCT shared by *A. coerulea* and *V. vinifera* (Figures 3A,B,M and Supplementary Tables 5, 10). Among all trees that support T1 (Figure 3A), 34.07% (31/91) trees support RCT shared by *A. coerulea* and *P. somniferum*, and only 19.78 (18/91) trees support the RCT occurred after the divergence of *A. coerulea* from *P. somniferum* (Figures 3C,D,N and Supplementary Tables 6, 10). This is a significant support if similar analysis of grass was considered, because 31–37% of duplicated gene trees in different species support a grass-common WGD event (Paterson et al., 2004). Some trees have inconsistent topologies likely caused by divergent evolutionary rates of recursively duplicated genes. Then, to examine the NST and MST events, 174 trees for orthogroups were constructed, which containing one pair of *N. nucifera* paralogous genes, one pair of *M. integrifolia* paralogous genes, and at least one *T. sinense* and *V. vinifera* orthologous genes to the *N. nucifera* and *M. integrifolia* genes. A total of 81.03% (141/174) and 72.41% (126/174) trees support that the NST and MST are lineage-specific events (Figures 3E,F,O and Supplementary Tables 7, 10). Similarly, 192 trees of orthogroups were constructed, each of which have at least three paralogous genes of *T. sinense*, and one *N. nucifera, A. coerulea*, and *V. vinifera* orthologous genes. Eventually, 46.88% (90/192) trees support two *Tetracentron*-specific WGD events (TAT and TRT) (Figures 3H,P and Supplementary Tables 8, 10). Moreover, using the orthogroups which involving three ECH-related paralogous genes and at least one *T. sinense, N. nucifera*, and *A. coerulea* orthologous genes, we constructed 163 trees. Notably, 40.49% (66/163) trees likely support the ancestor of *A. coerulea* donated genes for the ECH-related paralogues (Figures 3J,Q and Supplementary Tables 9, 10).

### Multiple Genome Alignment to Construct a Framework for Eudicots

Using the *V. vinifera* genome as the reference, a multiple alignment table was established to store homologous information between and within the genomes (Supplementary Table 11). We filled all *V. vinifera* gene identifiers in the first column of the table and added the gene identifiers of other species, column by column, and species by species, according to the colinear relationships inferred by multiple alignments. If no gene loss occurs, each *V. vinifera* gene has two orthologous genes in the *N. nucifera, M. integrifolia, A. coerulea*, and *A. oxysepala* and four orthologous genes in *P. somniferum* and *T. sinense* genomes, respectively. When a gene was absent in the expected location (usually due to gene loss, translocation, or inadequate assembly), this is recorded in the corresponding cell. Finally, the multiple alignment table including 19 = (3+2+2+2+2+4+4) columns contains three subgenomes of *V. vinifera*, two subgenomes of *N. nucifera, M. integrifolia, A. coerulea*, and *A. coerulea*, and four subgenomes of *P. somniferum* and *T. sinense*, respectively. The table summarizes the results of multiple genome and event-related alignments and shows the paralogous genes (or chromosomal regions) produced by genome triplication and duplication, as well as orthologous genes (or chromosomal regions) established by species (Supplementary Figure 11). However, the multiple alignment table with *V. vinifera* genome as the reference cannot reflect the complete information of homologous collinearity. In particular, it cannot contain all the duplicated genes that are produced by specific WGD of basal eudicots. In other words, genes colinear to the *V. vinifera* genes in the basal eudicots may be lost. Therefore, we constructed another table with *A. coerulea* genome as the reference, as a complement to Supplementary Table 11 to better represent the composition of eudicot genes and to establish a framework for other eudicots (Supplementary Figure 12 and Supplementary Table 12).

**Figure 4:**
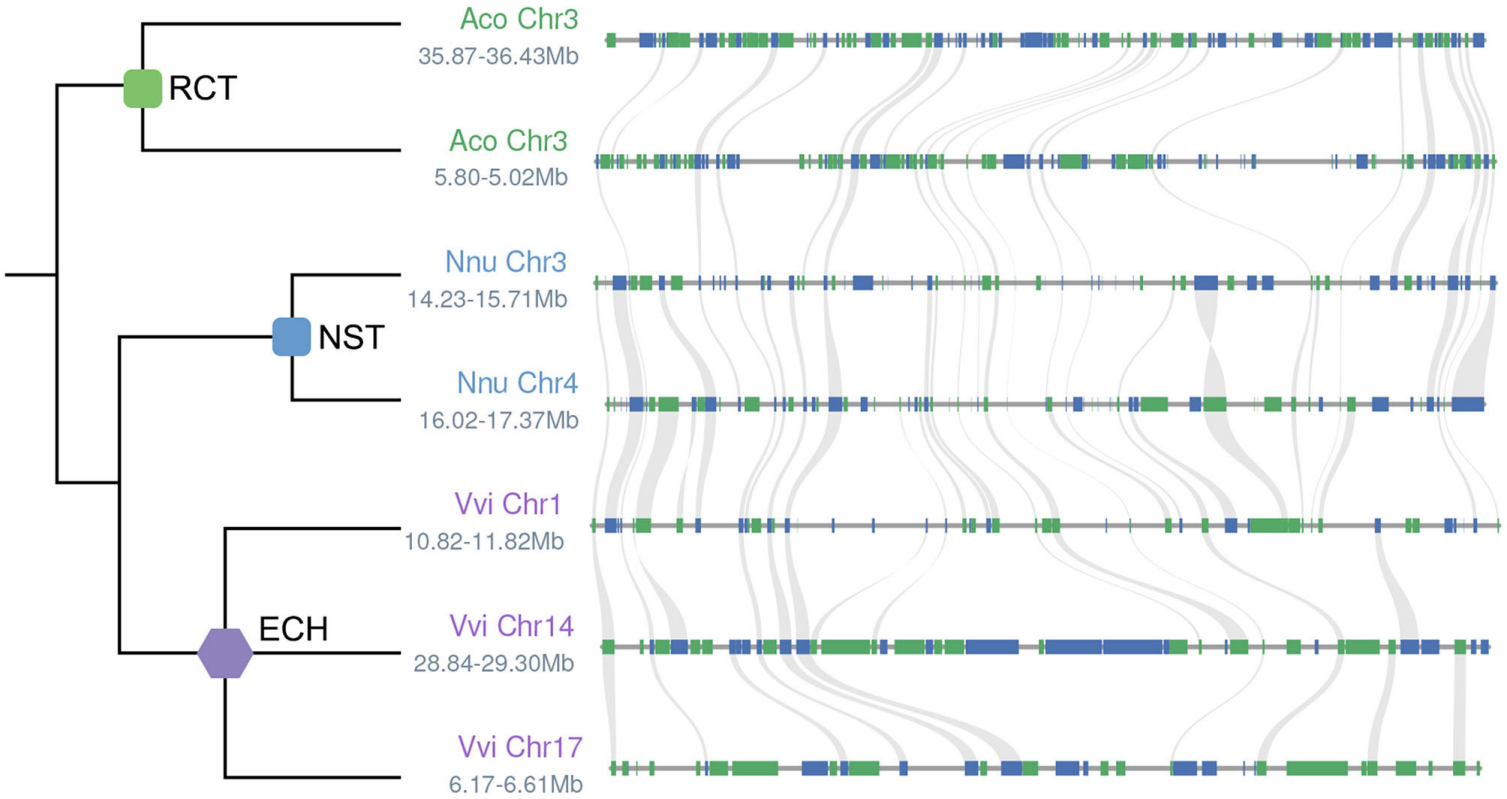
Local alignment in *A. coerulea, N. nucifera*, and *V. vinifera* genomes. The phylogeny of homologous regions is shown on the left. The chromosomes of *A. coerulea* (Vvi), *N. nucifera* (Nnu), and *V. vinifera* (Vvi) and the locations on chromosomes are shown on the left side of the chromosome segments. Genes are represented as rectangles and homologous genes between adjacent chromosomal regions are linked with gray curves.

Any local alignment at the genome level can use linear relationships to clearly show homology of chromosomal regions within and between genomes, and to see in detail the occurrence of gene loss or gene translocations. We selected local alignment regions with *A. coerulea* as the reference (Figure 4). A pair of paralogous regions generated by RCT in the *A. coerulea* genome were used. They correspond to two orthologous regions in *N. nucifera* and three in *V. vinifera*. In the local alignment regions, it can be discerned that some genes have colinear genes in each selected local chromosomal region, that is, some homologous genes are present in all expected chromosomal regions after species divergence or after WGD. However, there are a large number of genes without colinear genes at their expected locations on the corresponding chromosomes, revealing that widespread gene loss or translocation occurred in their ancestral genomes.

### Evolutionary Divergence and Dating

The kernel function analysis is used to distinguish different *Ks* distributions, and then, the main normal distributions in each observed *Ks* distribution are evaluated by linear combinations of several normal distribution functions. Eventually, each identified *Ks* peak is associated with an ancient evolutionary event (polyploidization or speciation) (Figure 1A and Supplementary Table 3). According to previous studies, the time of divergence between *V. vinifera* and *N. nucifera* was estimated to be ~125–135 Mya (Moore et al., 2010), and the gamma event occurred at ~115–130 Mya (Jiao et al., 2012). However, before evolutionary rate correction, the peak value of *Ks* between *N. nucifera* and *V. vinifera* is ~0.934 (±0.104), which is younger than the peak value of *Ks* in the *V. vinifera* genome is ~1.029 (±0.244), mainly because the evolutionary rate of *N. nucifera* is slower than *V. vinifera*.

To determine the age of polyploidization and speciation for selected species, we developed a model to correct diverged evolution rates (refer to Supplementary Text for details). After correction, the *Ks* peak between *A. coerulea* and *N. nucifera* or *V. vinifera* ~1.072 (±0.127) (Supplementary Table 4) corresponds to the ancestral divergence of *A. coerulea* and *V. vinifera*/*N. nucifera*. Based on the occurrence time of gamma ~115–130 Mya (Jiao et al., 2012), we deduced that the divergence time of *A. coerulea* and *N. nucifera*/*V. vinifera* was ~123–139 Mya (Figures 1B,C). The NST occurred at ~58–66 Mya, close to the previously reported time of ~54–74 Mya (Ming et al., 2013), at the K-P boundary. The RCT event shared by *A. coerulea* and *P. somniferum* was slightly later than the divergence of *A. coerulea* and *V. vinifera* or *N. nucifera*, occurring at ~118–134 Mya. Not long after the RCT event, divergence of *A. coerulea* and *P. somniferum* occurred at ~116–131 Mya, and the PST event was at ~9**–**10 Mya (Figures 1B,C). Notably, after correction, the *Ks* peak of *N. nucifera*-*V. vinifera* divergence at 0.934 (±0.104) was still younger than ECH peak at 1.000 (±0.238), implying that some sister groups of core eudicots (like the ancestor of *A. coerulea*) could donate the genes to ECH hexaploid.

### Genomic Fractionation

With *V. vinifera* genome as the reference, it is clear that extensive genomic fractionation occurs after WGD events (NST and RCT). For example, using *V. vinifera* chromosome 1 as the outgroup, 61 and 57% of *V. vinifera* genes were not found at the respective colinear locations in *N. nucifera* duplicated regions generated by NST, and 47% of *V. vinifera* genes had no colinear genes in *N. nucifera* expected duplicated regions (Supplementary Table 13). With *V. vinifera* chromosome 1 as the reference, more gene loss or translocation occurred in each or both subgenomes of *A. coerulea* produced by RCT (Supplementary Table 14). Using the *A. coerulea* genome as the reference, *N. nucifera* and *V. vinifera* genome fractionation were also explored (Supplementary Tables 15, 16), and extensive genomic fractionation occurs after WGD events (NST and ECH).

**Figure 5:**
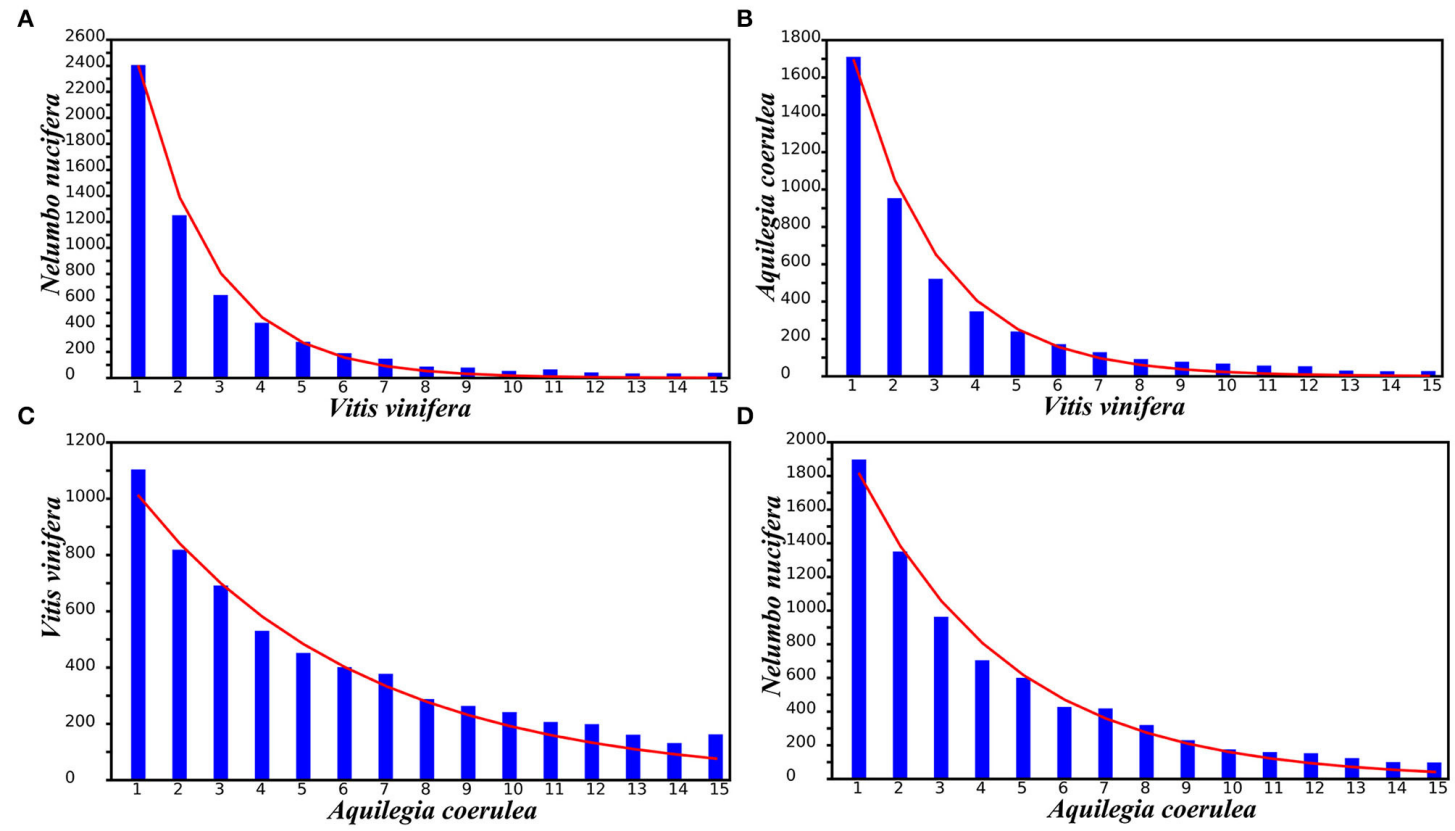
Geometric distributions of gene loss rates fitting: **(A)** *N. nucifera* to the *V. vinifera* genome, **(B)** *A. coerulea* to the *V. vinifera* genome, **(C)** *V. vinifera* to the *A. coerulea* genome, and **(D)** *N. nucifera* to the *A. coerulea* genome. The x-axis indicates numbers of continuously missing genes in gene collinearity regions.

The amount and length of gene loss or translocation may occur in an almost random manner and can be described by an extremely approximate geometric distribution. Taking the *V. vinifera* genome as the reference, the distributions of different gene loss regions observed in *N. nucifera* and *A. coerulea* were fitted by using geometric distribution curves of different densities (Figures 5A,B and Supplementary Table 17). In addition, taking *A. coerulea* as the reference, the distributions of different gene loss regions observed in *V. vinifera* and *N. nucifera* were fitted (Figures 5C,D and Supplementary Table 17). Comparing the difference in geometric distribution curves between species, we found that the longer of genes were lost, the greater of deviation between the observed number and the number predicted by theory. To reveal the potential mechanisms and scales of genomic fractionation, we counted the number of continuously removing genes at the colinear locations of each genome corresponding to the reference genome. Most of the removing genes constitute “small runs,” with one or two removing genes in succession (Figure 5). As a mutual reference, we compared the small runs of *V. vinifera* and *A. coerulea* and found that *A. coerulea* has more small runs, suggesting that there may be more recent (or ongoing) gene loss in *A. coerulea* to destroy genomic collinearity.

### Unbalanced Fractionation Between the WGDs Homoeologous Regions

#### Three Unbalanced *V. vinifera* Subgenomes

By comparing the duplicated regions of the *V. vinifera* genome corresponding to each chromosome in *A. coerulea*, we identified three sets of paralogous chromosomes generated by ECH and found similar gene loss or transfer rates (Supplementary Table 15). With each of the seven chromosomes of *A. coerulea* as the reference, respectively, the differences in gene loss rates of paralogous regions in *V. vinifera* are <0.05, and the mean difference in loss rates is 0.02. When using chromosomes 5 and 7 of *A. coerulea* as the reference, there are paralogous regions in *V. vinifera* with almost identical gene loss rates. The greatest difference in gene loss rate between the paralogous regions of *V. vinifera* is 0.04. It seems that paralogous chromosomes generated by ECH have balanced gene loss rates, consistent with previous findings suggesting no significant differences in gene retention between duplicated chromosomal regions (Tang et al., 2008). However, detailed studies of gene retention and loss level using sliding windows along chromosomes suggest that regions of duplicated chromosomes may originate from three unbalanced subgenomic divergences. Aligned with the reference genome, local regions show variation in gene retention ~0–31.68% (Supplementary Figure 13).

We further compared the retention levels of the three *V. vinifera* subgenomes (A1, A2, and A3) generated by ECH according to one gene as a step with a sliding window of 100 genes. Using the *N. nucifera* genome as the reference, there were only 58.34, 58.22, and 65.53% homologous sliding windows for A1-A2, A1-A3, and A2-A3, respectively, showing no significant difference (less than 5% difference in gene retention rates: *p* < 0.05) in genomic fractionation (Supplementary Table 18). Then, we employed the recently proposed polyploidy index (P-index) as a mathematical quantitative indicator to infer the evolutionary types of polyploids (Wang et al., 2019a). Based on phylogenetic inference, *N. nucifera* is relatively close to *V. vinifera*, so we chose the *N. nucifera* genome as the reference, yielding a P-index of 0.46 for ECH (Supplementary Table 19). It supports that the three subgenomes generated by ECH are divergent, which shows an allopolyploid nature.

#### Two Unbalanced *N. nucifera* Subgenomes

With *V. vinifera* chromosomes as the reference, we compared the paralogous regions in *N. nucifera* produced by NST and found nine chromosomes with gene loss rates difference > 0.05 (Supplementary Table 13). Using seven *A. coerulea* chromosomes as the reference, we found that no paralogous chromosomes in *N. nucifera* have a gene loss rate difference > 0.05 (Supplementary Table 16). Perhaps using the whole chromosomes from reference genome to compare the gene loss rate, there is no significant difference between homologous regions. Their difference may be hidden. Thus, we also used sliding windows along chromosomes to identify the balance of duplicated chromosome regions gene retention and loss level. It is found that the regions of duplicated chromosomes may originate from two unbalanced subgenomic fractionation. Aligning with the reference genome, the local regions show a change in gene retention rates ~0–69.31% (Supplementary Figures 14A,B). Further, with the *V. vinifera* genome as the reference, we compared the retention levels of the two *N. nucifera* subgenomes generated by NST under the same criteria and found that the window with no significant difference was only 39.13% (*p* < 0.05) (Supplementary Table 18). The P-index for NST was calculated as 0.52 (Supplementary Table 19), with *V. vinifera* as the outgroup, which also demonstrates the allopolyploid nature.

#### Two Unbalanced *A. coerulea* Subgenomes

**Figure 6:**
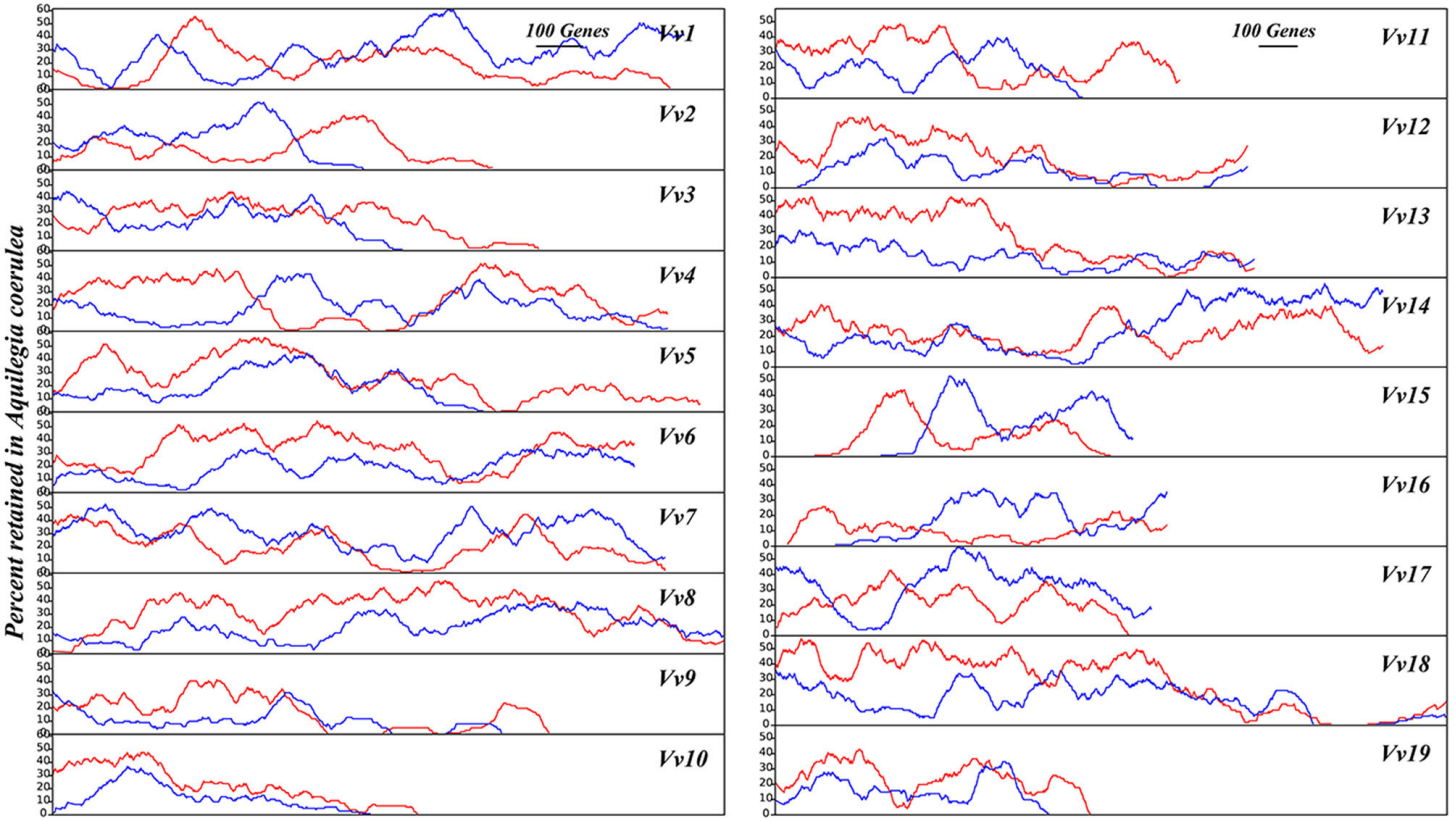
*A. coerulea* gene retention along corresponding orthologous *V. vinifera* chromosomes. Using the *V. vinifera* chromosomes as the reference, with 100 genes as a sliding window, the percentage of gene retention in two sets of *A. coerulea* subgenomes is shown in red and blue lines, respectively.

Similarly, with *V. vinifera* chromosomes as the reference, we found that the two duplicated regions of *A. coerulea* produced by RCT often have divergent gene loss rates. For 17 of the 19 *V. vinifera* chromosomes, the gene loss rate difference between two *A. coerulea* duplicated regions is >0.05 (Supplementary Table 14). We also used sliding windows to show the unbalanced gene retention level, revealing that the *A. coerulea* have two unbalanced subgenomic fractionation (Figure 6). Aligning with the reference genome, the local regions show a change in gene retention rates ~0–69.39%. Furthermore, with the *N. nucifera* genome as the reference, we compared the retention levels of the two *A. coerulea* subgenomes generated by RCT under the same criteria and found that the window with no significant difference was only 37.67% (*p* < 0.05) (Supplementary Table 18). With *N. nucifera* as the outgroup, we calculated the P-index for RCT as 0.39 (Supplementary Table 19). However, considering that *N. nucifera* genome has more specific chromosomal rearrangements (Gui et al., 2018), the *V. vinifera* genome was taken as the additional reference, and the P-index for RCT is 0.66, suggesting that the allopolyploid nature of RCT event.

## Discussion

### Polyploidization Events During the Early Diversification of Eudicots

Recursive polyploidy is common in plants and it is the key driving force for species diversification, contributing to genetic innovation and adaptation to extreme environments (Jiao et al., 2011; Van de Peer et al., 2017; Landis et al., 2018). With the completion of genome sequencing and resequencing, many WGD events have been identified, such as the 1,000 transcriptome sequencing analysis, which identified 244 WGDs at different locations in the phylogeny of green plants at one time (Leebens-Mack et al., 2019). However, the timing of WGDs occurring at rapidly diverging nodes remains a huge challenge as shown by several recent studies which generated controversial results regarding WGDs that occur in the early stage of rapid eudicot diversification. The central question is whether there was a shared WGD event in all eudicots (Aköz and Nordborg, 2019, 2020; Liu et al., 2020; Shi and Chen, 2020). Here, we integrated genomic structure, phylogenetic, and *Ks* distribution analysis and performed genomic hierarchical and event-related alignments to infer homology between and among representative species of the eudicots. The results strongly suggest that there was no shared WGD across all eudicots. The paleohexaploid resulting from the gamma event (ECH) is the only common ancestor of the core eudicots. Indeed, the WGD (RCT) in *A. coerulea* is particularly old and is shared with *P. somniferum*, almost at the base of eudicots divergence, but not shared by Proteales.

Eudicot plants appeared on Earth about 130–150 Mya (Jiao et al., 2011; Magallon et al., 2015) and the sister groups of core eudicots diverged from the core eudicots in a relatively short period, maybe within only several million years (Bell et al., 2010; Magallon et al., 2015). As shown above, we dated the RCT and ECH events as occurring independently, and they are close to the divergence of Ranunculales from other eudicots. In addition, the *A. coerulea*–*P. somniferum* divergence occurred shortly after the RCT. The evolutionary events occurring at rapid diverging nodes present a challenge in terms of timing them accurately, mainly due to the limited extent of DNA sequence divergence information retained by existing species. As a result, most of the gene trees obtained from phylogenetic analysis do not have sufficient resolution to effectively support the judgment of evolutionary relationships (Jiao et al., 2012; Chanderbali et al., 2017; Shi and Chen, 2020). Our previously developed pipeline which integrates genomic homologous dot plots, *Ks* distribution of colinear genes, and phylogenetic genomic analysis is an appropriate tool for these WGD events (Wang et al., 2018b). In addition, polyploidization often produced a large number of duplicated genes, complex chromosomal rearrangements, and duplicated gene loss (Soltis et al., 2015; Murat et al., 2017; Wang et al., 2017, 2018b, 2019b), which hinders the inference of polyploidy history, and can lead to problematic conclusions.

We found that the colinear depth ratio of *A. coerulea*–*P. somniferum* is 2:4 (Supplementary Figure 10). However, Shi and Chen proposed that the colinear depth ratio is 2:2 (Shi and Chen, 2020). This conflicts due to *P. somniferum* undergoing complex chromosome rearrangement, and a large amount of duplicated gene loss, resulting in that they only identified the recent WGD event (Guo et al., 2018; Shi and Chen, 2020). The ancient WGD of *P. somniferum* may have lost more duplicated genes than the recent WGD, thus mixing together the 4DTV peak of homologous genes produced by large- and small-scale genomic duplications. Moreover, sequencing analyses of CHM genome suggested that *P. somniferum* underwent two WGDs and the ancient one was shared with *A. coerulea*, and only the 4DTV peaks and paralog peaks are available as evidence to support this (Pei et al., 2021). Therefore, in addition to comparative genomic and phylogenetic analyses, we counted the RCT- and PST-produced colinear blocks in *P. somniferum*. They covered 60.69 and 82.39% of the genome, respectively, which is very similar to the two identified WGDs (ξ and η events) produced blocks of *Selaginella* reported in previous studies, covering 64.8 and 76.7% of the genome, respectively (for details, refer to Supplementary Text; Wang et al., 2020b). These comparisons supported that the two rounds duplication of *P. somniferum* are two whole-genome duplication events, rather than one WGD and one segmental duplication.

### Event-Related Alignment of Selected Eudicots

Identification of gene homology relationships in plant genomes is extremely difficult due to large-scale chromosomal reorganization following recurring polyploidy events (Soltis et al., 2014, 2015). After reconstructing the WGD events during the early diversification of eudicots, we selected representative species of eudicots for intragenomic and intergenomic comparisons and constructed multiple genome alignment of *A. coerulea, N. nucifera*, and *V. vinifera* associated with polyploidization and speciation. The constructed alignment was shown by a homologous gene list, including orthologous produced by species divergence and paralogous produced by ancestral polyploidization events. This list of homologous collinearity genes can provide a valuable genomic platform for researchers, to search for the functional genes (or gene families) which are related to the economically and agriculturally important traits and investigate the origin of genes, functional innovations, and the phylogenetic relationships of involving families and regulatory pathways in multiple species. Moreover, this event-related alignment provides a general framework for the alignment analysis of eudicots and others, and new genome aligning information could be convenient to add in this alignment.

### Gene Loss and Retention

Homologous chromosomal regions produced by polyploidization may have substantial gene loss and translocation. *A. coerulea, N. nucifera*, and *V. vinifera* retained only a small fraction of the duplicated genes generated by polyploidization after RCT, NST, and ECH events, respectively. Large-scale duplicated gene losses following polyploidization have been recognized (Long et al., 2003; Mitchell-Olds and Schmitt, 2006; Soltis et al., 2016). This phenomenon has been further confirmed in cotton (Wang et al., 2016), legumes (Wang et al., 2017), and Cucurbitaceae (Wang et al., 2018b). Genome fractionation accumulated by small runs of lost genes (Schnable et al., 2011; Wang et al., 2017) and the length of fractionated regions grows with time. This may reflect the direction of evolution to some extent and helps polyploids eventually become stable, but this hypothesis needs to be tested. With selected reference genomes, we found that gene loss in *V. vinifera, N. nucifera*, and *A. coerulea* occurred randomly and could almost be described by geometric distributions. However, the actual pattern of gene loss could be more complex, as there was an obvious deviation of the data from the random distribution. Natural selection, convergent evolution, and domestication could be changing the patterns of genes loss in certain regions, and this needs to be investigated in the future with appropriate methods.

### Divergent Genomic Evolutionary Rates

Here, the distributions of synonymous nucleotide substitutions at synonymous sites (*Ks*) of colinear genes were employed to compare the divergence levels of genomic evolutionary rates. We found the significantly divergent evolutionary rates among the considered species after the polyploidization and speciation events. This situation widely exists in many other angiosperm lineages, such as the families of Cucurbitaceae (Wang et al., 2018b), Fabaceae (Wang et al., 2017), Malvaceae (Wang et al., 2019b), Apiaceae (Wang et al., 2020a), and Poaceae (Wang X. et al., 2015). As previous studies demonstrated, the divergent evolutionary rates could be caused by the differentiation difference in duplicated genes produced by polyploidization (Soltis et al., 2015); for example, the maize genome has a faster evolution rate than other grasses, which may be driven by its specific WGD (Wang X. et al., 2015). Among the Ranunculales, *P. somniferum* evolved faster than *A. coerulea*, which perhaps influenced by the PST event. In addition, previous studies suggested that *N. nucifera* evolved slower than *V. vinifera*, pointing to the evolutionary rates may be influenced by the living environment of species (Ming et al., 2013; Wang et al., 2017). The high divergence evolutionary rates among different species may affect the evaluation of phylogenetic relationships and the age of evolutionary events, hindering our understanding of the origin and evolution of species and some genes with important trait. Therefore, we emphasize taking the evolutionary events with the same age shared by species as the correction benchmark, correcting the key polyploidization and speciation events other than the correction benchmark, and then infer the relatively accurate evolutionary of studied species.

### Hypothetical Paleo-Allopolyploidization

Depending on how polyploids are formed, they can be classified as autopolyploids and allopolyploids, mainly because of the different origins of the subgenomes. If the reports about the nature of polyploidy are relatively accurate, allopolyploidy may be more common in the process of plant evolution, especially in favor of the emergence of large plant groups (Ramsey and Schemske, 2002). Previous studies suggested that *V. vinifera* and *N. nucifera* may be allo-paleopolyploid (Aköz and Nordborg, 2019; Shi et al., 2020). Here, we confirmed again by the genomic fractionation patterns between subgenomes in *V. vinifera* and *N. nucifera*. This also demonstrated that the genomic fractionation of allopolyploid and autopolyploid shows two different states of imbalance and balance, respectively, and the robustness of P-indices for “diagnosing” the nature of polyploidies (Wang et al., 2019a). Using the similar method, we inferred the likely allopolyploid nature of RCT event.

Allopolyploids have a heterosis and higher offspring survival rates (Ramsey and Schemske, 2002), which may provide more opportunities for plant diversity, and our study also supports the role of allopolyploidy in speciation of early clades of eudicots. Thereby, the three allopolyploidy (ECH, NST, and RCT) events are associated with the establishment of the major clades of eudicots. The divergence between *V. vinifera* and *N. nucifera* is still earlier than the ECH event after evolutionary rate correction (Figure 1B), which may be due to the donation of *A. coerulea* to *gamma* hexaploidy. Additionally, the results of phylogenetic analysis also seem to support this hypothesis, as the previous studies proposed “two-step duplication” model, the sister group (likely ancestor of *A. coerulea*) of core eudicots hybridized with the ancestor of core eudicots to form *gamma* hexaploid (Ming et al., 2013; Chanderbali et al., 2017). This is different from the recent hypothesis that gamma hexaploidy originated from the hybridization of ancestral tetraploid of all eudicots and one diploid (Aköz and Nordborg, 2019). Nevertheless, we are still not confident enough to determine the origin of *gamma* hexaploidy, because it remains hardly difficult to exclude the result of reticular hybridization, partial genomic introgression, parallel evolution, and incomplete lineage sorting (ILS) during the early diversification of eudicots.

## Materials and Methods

### Materials

We downloaded genomic sequences and annotations from the relevant websites of genome projects, for which complete information can be found in Supplementary Table 22.

### Inferring Gene Collinearity

Gene collinearity indicates that the genes and gene order among genomes are conservative to some extent, which can reflect the homologous chromosomal structure of the shared ancestor; it is essential for understanding genomic changes, especially in inferring the evolution of complex plant genomes. First, protein sequences are searched for potential homologous genes (*E* < 1e-5) by performing BLASTP (Altschul et al., 1990). Second, gene homology information was used as input to ColinearScan (Wang et al., 2006) to locate colinear gene pairs. The key parameter, the maximum gap, was set at 50 intervention genes as per the approach taken in previous genomic research (Wang et al., 2018b). We compared *A. coerulea, N. nucifera*, and *V. vinifera* genomes and identified the intragenomic and intergenomic colinear genes. Colinear genes preserved in modern genomes can provide important genetic homologous imprints to reveal ancient evolutionary events.

### Synonymous Substitutions

Using the Bioperl Statistical module, synonymous nucleotide substitutions on synonymous sites (*Ks*) were estimated through the Nei–Gojobori approach (Nei and Gojobori, 1986).

### Identification of the Genomic Homology and the Polyploidy-Produced Subgenomes

To identify the genomic homology including paralogy within genomes and orthology between genomes, we performed the genome colinear dot plots integrated with the *Ks* medians of colinear gene pairs in homologous chromosomal regions (Supplementary Figures 3–10). The orthologous and paralogous chromosomal regions were identified by comparing the *Ks* values of colinear regions related to the species divergence and specific polyploidy events. When identifying subgenomes generated by polyploidization, a genome unaffected by that polyploidy event is required as a reference and the reference genome has good assembly quality. There are fragmented orthologous regions between the reference genome and the considered genome, and these orthologous regions can be sequentially assigned to different subgenomes. The chromosomes with higher gene losses were inferred to be from a sensitive subgenome, whereas the others from a dominant subgenome (Schnable et al., 2011). If the reconstructed chromosomes show no difference in gene loss, they were assigned arbitrarily to each subgenome.

### Calculation of the P-Index

To infer the possible nature of polyploids, the degree of divergence among polyploid subgenomes was estimated by previously developed statistics, the polyploidy-index (P-index) (Wang et al., 2019a). The P-index value would fall in the range of [0–1]. When the value is infinitely close to 0, it indicates that there is almost the same genomic fractionation level between subgenomes; on the contrary, when the value is infinitely close to 1, it shows that one of the subgenomes has an absolutely fractionation advantage. Previous studies have demonstrated that the robustness of P-indices for “diagnosing” the nature of polyploidies, which is supported by considering the P-indices > 0.3, including several known or previously inferred paleo-allopolyploids of *Brassica napus, Zea mays, Gossypium hirsutum*, and *Brassica oleracea* (Schnable et al., 2011; Chalhoub et al., 2014; Li et al., 2014; Wang M. et al., 2015; Renny-Byfield et al., 2017), whereas the small values point to several paleo-autopolyploids of *Glycine max, Populus trichocarpa*, and *Actinidia chinensis* (Liu et al., 2017; Wang et al., 2017, 2018a). Therefore, we could infer the evolutionary types of polyploids and assess their evolutionary impact based on a P-index threshold of 0.3. The detailed P-index calculation scheme here is as follows.

First, when calculating P-index, a reference genome was employed to infer orthologous regions with the studied genome, and identifying the potential gene losses or translocations in each of the inferred subgenomes produced by an ancient polyploidization event. A well-assembled, rare specific genomic rearrangement, and evolutionarily close genome could serve as an ideal reference genome for estimating the p-index value of considered genome. For example, using *N. nucifera* and *V. vinifera* as reference genomes, respectively, the p-index of RCT events was calculated. *N. nucifera* is evolutionarily close to Ranunculus, and *V. vinifera* has less genome rearrangement. Then, the subgenomes of a considered polyploidy-affected genome were mapped onto a selected reference genome, which is not affected by the polyploidization event. Assuming that there were *K* chromosomes in the reference genome, the subgenomes A and B identified in the considered genome. Regardless of whether one dominates, each pair of homoeologous chromosomes was divided into *N*_*C*_ Windows with *M* (such as 100) genes. For the *i*-th window of a specific homoeologous chromosome pair, the gene retention rate *A*_*i*_ and *B*_*i*_ relative to the reference genome was obtained, and thus, the P-index value was conferred as follows:


$$\begin{array}{l} P-index=\sum_{C=1}W_{C}abs\left[\frac{\sum_{i=1}^{N_{C}}\frac{A_{i}-B_{i}}{abs\left(A_{i}-B_{i}\right)}\times \delta_{i}}{N_{C}-\delta \left(N_{C}\right)}\right], \end{array}$$

which would fall in the range of [0–1]. The shorter chromosomes in reference genome retain fewer colinear genes, leading to greater volatility. Thus, each chromosome is estimated with:


$$\begin{array}{l} W\text{c=}\frac{Nc}{\underset{i=1}{\sum }Ni}\left(c=1,2,\ldots ,K\right). \end{array}$$

Windows with similar or excessively different gene retention rates would affect the evaluation. To remove them, the evaluation coefficient was defined as follows:


$$\delta_{i}=\left\{ \begin{array}{l} 1,if\;0.1<d_{i}<3n\\ 0,if\;d_{i}\leq 0.1\text{ or }d_{i}\geq 3; \end{array}\right.$$

in which the gene retention difference level is defined as follows:


$$\begin{array}{l} di=abs\left[\frac{Ai-Bi}{\left(Ai+Bi\right)\times 0.5}\right]. \end{array}$$

### Topology Tree Construction

To clarify the WGD events of selected genomes, we used MEGA X (Kumar et al., 2018) to construct topology trees with homologous genes from multiple genome alignment lists. The maximum likelihood algorithm was employed to construct the gene trees using nucleotide sequences, and the bootstrap value was set to 1,000 to test the stability of the constructed gene trees, the gap was set as “Use all sites,” and the model was set as “Tamura-Nei model.” Based on the multi-genomic alignment homologous gene lists, we selected specific collinearity-based orthogroups to verify considered polyploidization events, respectively. The orthogroups selected to construct the gene trees should include those paralogues generated from the specific WGD events to be examined, as much as possible that the genes are generated from same ancestral chromosomes of considered genomes, excluding lineage-specific genes (selected orthogroups for inferring polyploidization events are described in Phylogenomic Analysis). In addition, some homologous genes were selected from other species that have not experienced the polyploidy events to be verified. Finally, for different evolutionary events, we constructed the gene trees. The independent or shared polyploidy events can be determined by the phylogenetic position of paralogous genes in the topology trees.

### Other Methods

Description of details about the “Kernel function analysis of *Ks*” and “Evolutionary rate correction” can be found in the “Supplementary Text.”

## Data Availability Statement

The original contributions presented in the study are included in the article/Supplementary Material, further inquiries can be directed to the corresponding author/s.

## Author Contributions

JinW conceived and led the research. JT implemented and coordinated the analysis. CW, SS, QX, YY, and YH performed the analysis. JianW, WG, JT, JinW, and LZ wrote the paper. All authors contributed to the article and approved the submitted version.

## Funding

We appreciate the financial support from the China National Science Foundation (31501333 and 32170236 to JinW) and the Natural Science Foundation of Hebei Province (C20209064 and C2015209069 to JinW).

## Conflict of Interest

The authors declare that the research was conducted in the absence of any commercial or financial relationships that could be construed as a potential conflict of interest.

## Publisher's Note

All claims expressed in this article are solely those of the authors and do not necessarily represent those of their affiliated organizations, or those of the publisher, the editors and the reviewers. Any product that may be evaluated in this article, or claim that may be made by its manufacturer, is not guaranteed or endorsed by the publisher.
