# Supplementary material for: Paleopolyploidies and Genomic Fractionation in Major Eudicot Clades
Source: Front Plant Sci. 2022 May 31;13:883140. doi: 10.3389/fpls.2022.883140 (PMC9194900; doi:10.3389/fpls.2022.883140)
Supplement: Supplementary file 1 [file Data_Sheet_1.PDF]

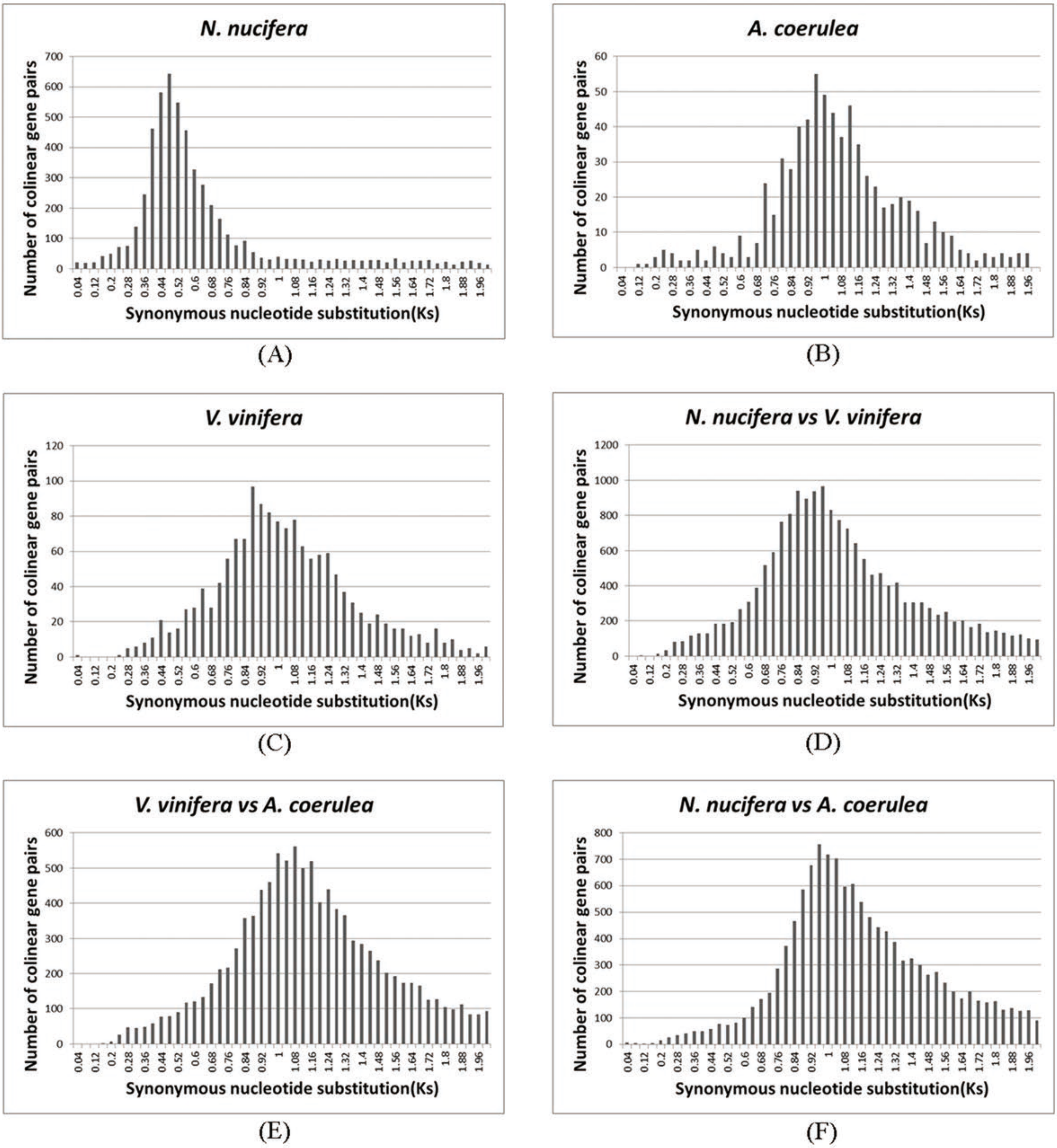

Figure S1. Histogram of the *Ks* distribution within and among genomes. The horizontal axis represents the *Ks* value from small to large, and the vertical axis represents the number of colinear gene pairs (**A**) within the lotus genome, (**B**) within the columbine genome, (**C**) within the grape genome, (**D**) between the grape and lotus genomes, (**E**) between the grape and columbine genomes, and (**F**) between the lotus and columbine genomes.

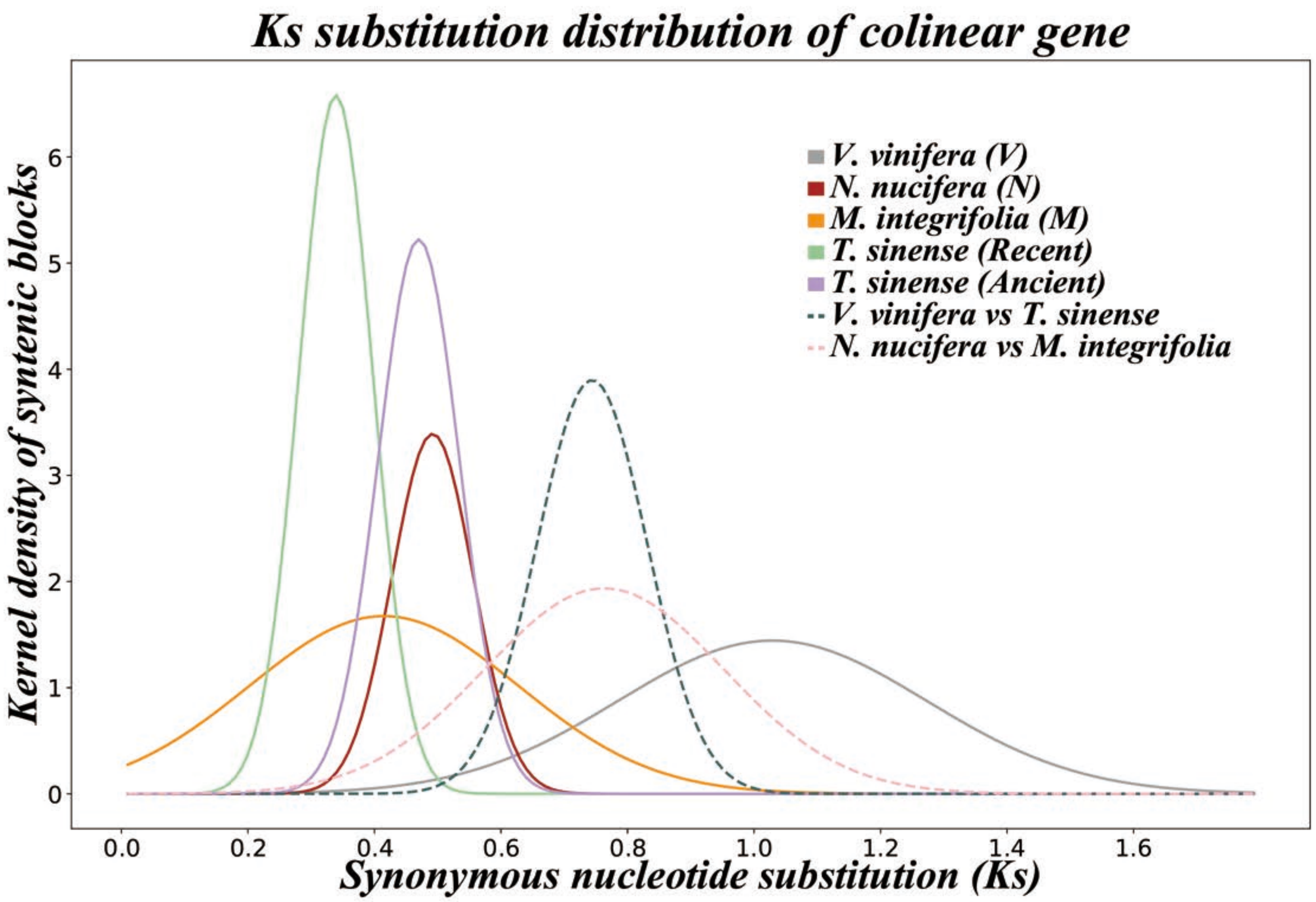

Supplementary Figure 2. *Ks* distribution of colinear genes within and among genomes. Grape (V), Lotus (N), Macadamia nut (M), Tetracentron (T). *Ks* distribution of colinear genes between (dashed curves) and within (solid curves) genomes.

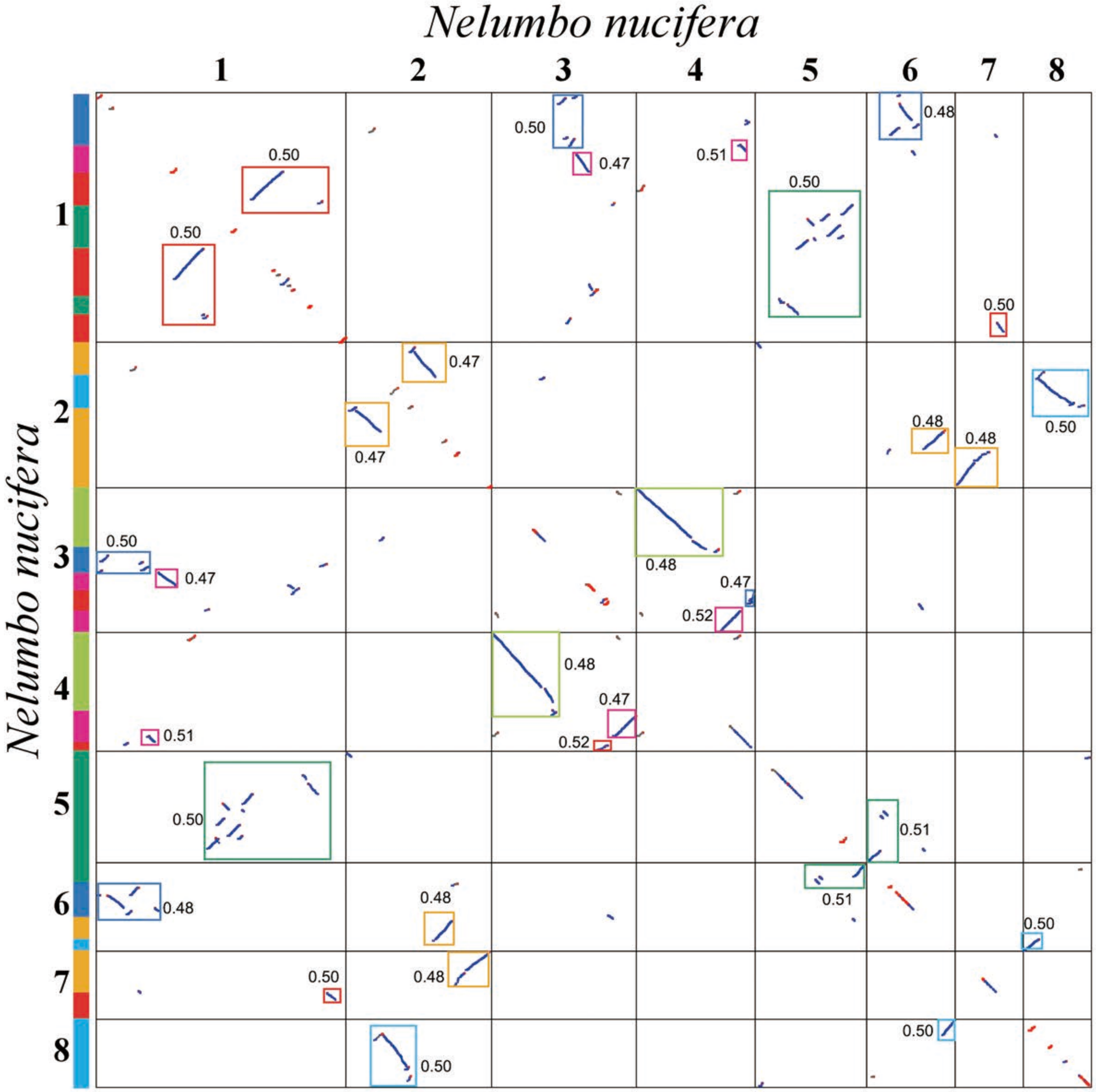

Supplementary Figure 3. Intragenomic homologous structure comparison analyses within the lotus genome. The best, secondary, and other matched homologous gene pairs output by Blast are plotted in red, blue, and gray respectively. The dotplot shows only relatively large paralogous regions (containing more than 8 gene pairs) in the lotus genome. The 8 lotus chromosomes are colored by the 7 eudicot ancestral chromosomes (Jaillon, et al. 2007). Paralogous regions within the genome are framed by solid line boxes of corresponding colors and the *Ks* medians of the regions are specified near the homologous gene regions.

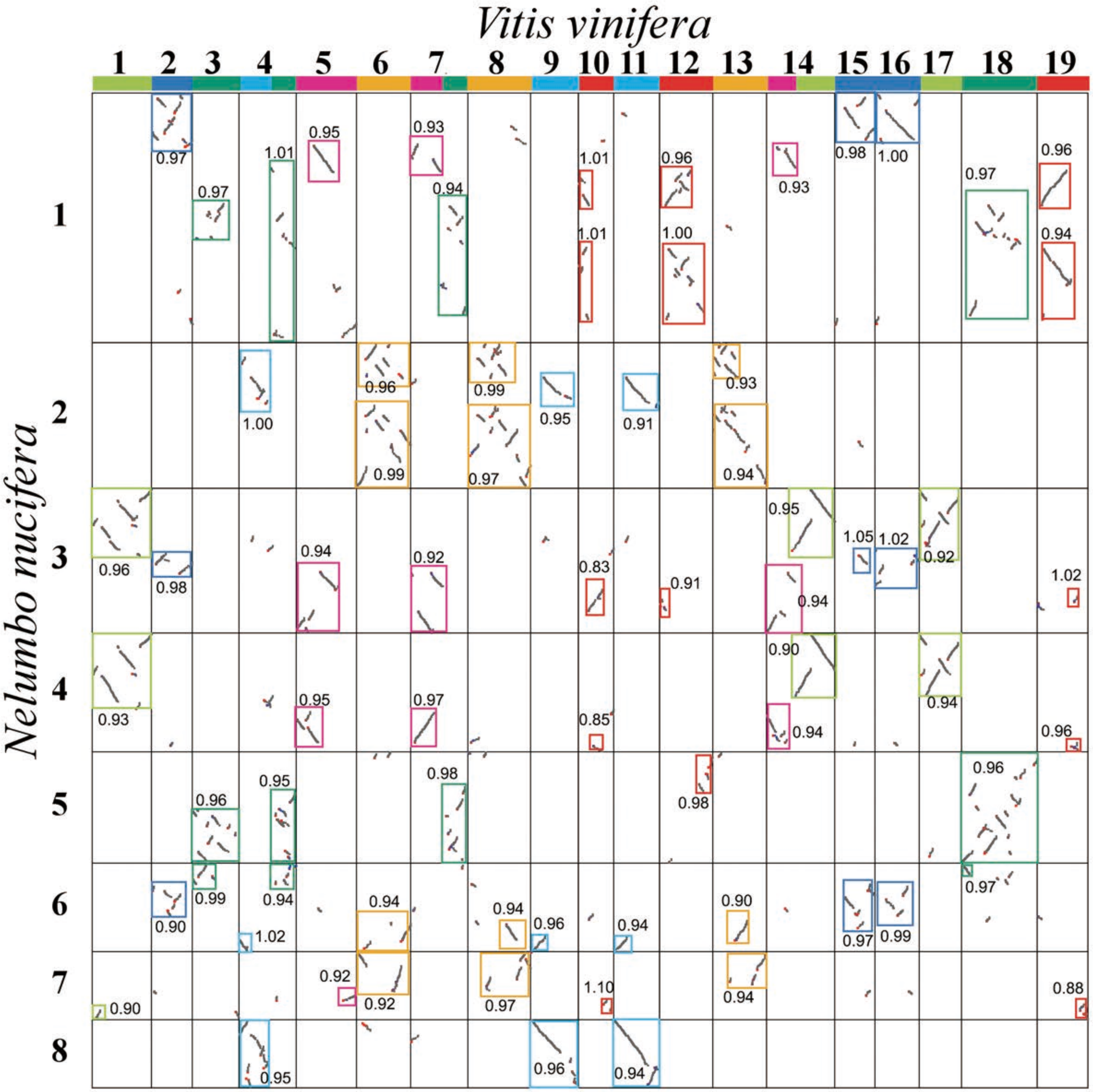

Supplementary Figure 4. Intergenomic homologous structure comparison analyses between the grape and lotus genomes. The best, secondary, and other matched homologous gene pairs output by Blast are plotted in red, blue, and gray respectively. The dotplot shows only relatively large orthologous regions (containing more than 8 gene pairs) between the grape and lotus genomes. The 19 grape chromosomes are colored by the 7 eudicot ancestral chromosomes (Jaillon, et al. 2007). Orthologous regions between the genomes are framed by solid line boxes of corresponding colors, and the *Ks* medians of the regions are specified near the homologous gene regions.

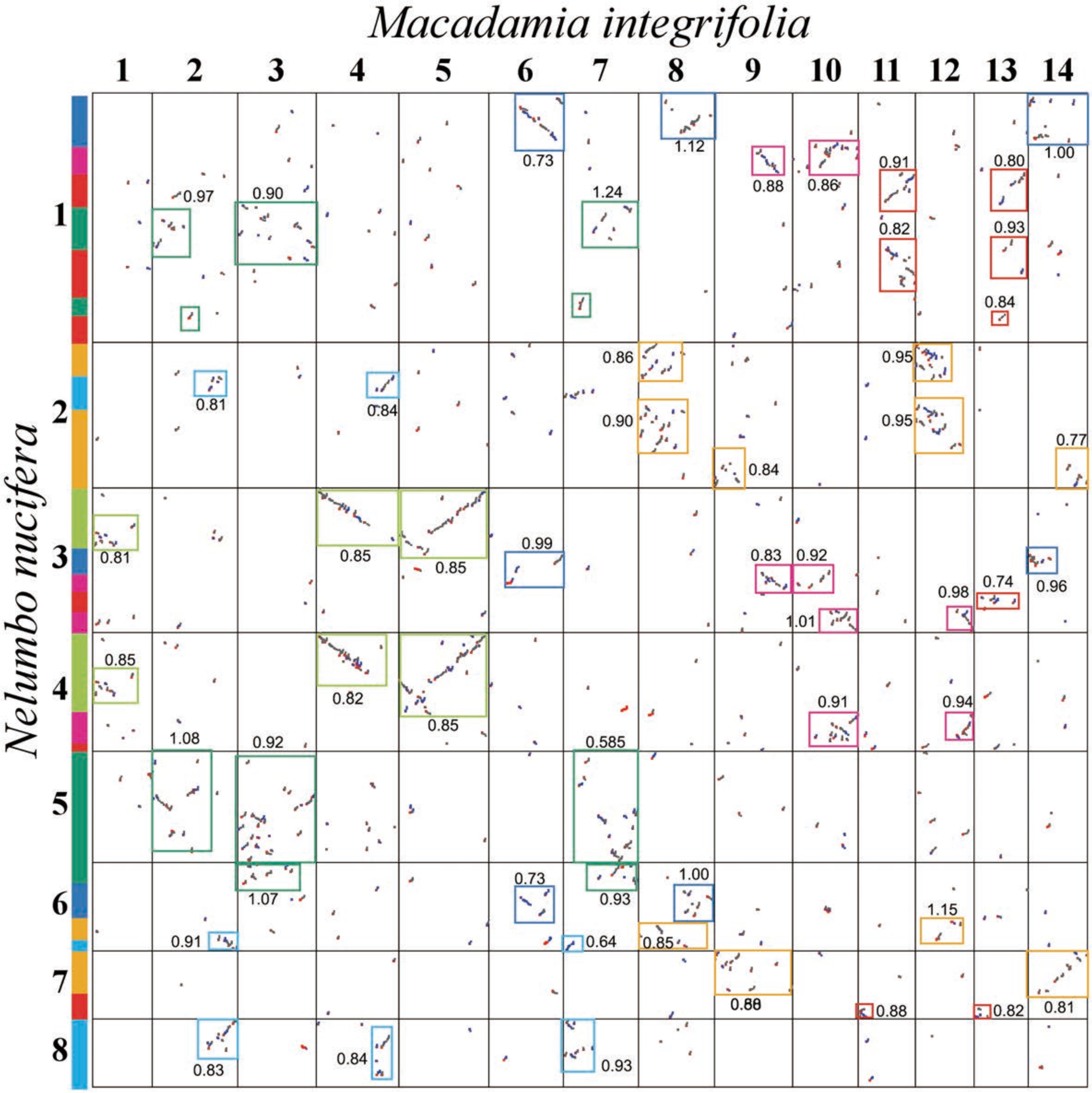

Supplementary Figure 5. Intergenomic homologous structure comparison analyses between the lotus and macadamia nut genomes. The best, secondary, and other matched homologous gene pairs output by Blast are plotted in red, blue, and gray, respectively. The dotplot only shows relatively large orthologous regions (containing more than 8 gene pairs) between lotus and macadamia nut genomes. The 8 columbine chromosomes are colored by the 7 eudicot ancestral chromosomes (Jaillon et al., 2007). Orthologous and paralogous regions between the genomes are framed by solid and dashed line boxes of corresponding colors, respectively and the *Ks* medians of the regions are specified near the homologous gene regions.

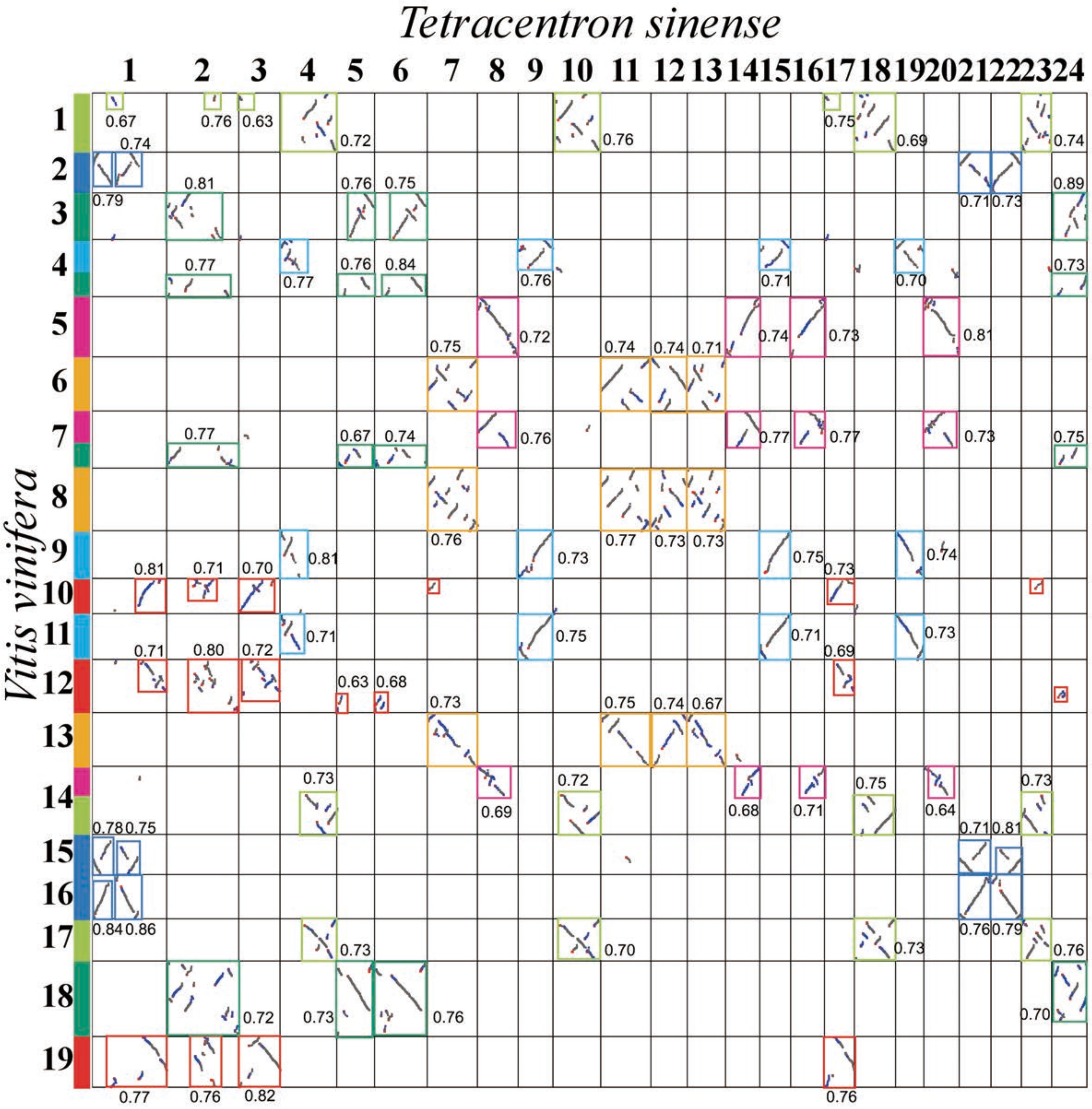

Supplementary Figure 6. Intergenomic homologous structure comparison analyses between the grape and tetracentron genomes. The best, secondary, and other matched homologous gene pairs output by Blast are plotted in red, blue, and gray, respectively. The dotplot only shows relatively large orthologous regions (containing more than 8 gene pairs) between grape and tetracentron genomes. The 19 grape chromosomes are colored by the 7 eudicot ancestral chromosomes (Jaillon et al., 2007). Orthologous and paralogous regions between the genomes are framed by solid and dashed line boxes of corresponding colors, respectively and the *Ks* medians of the regions are specified near the homologous gene regions.

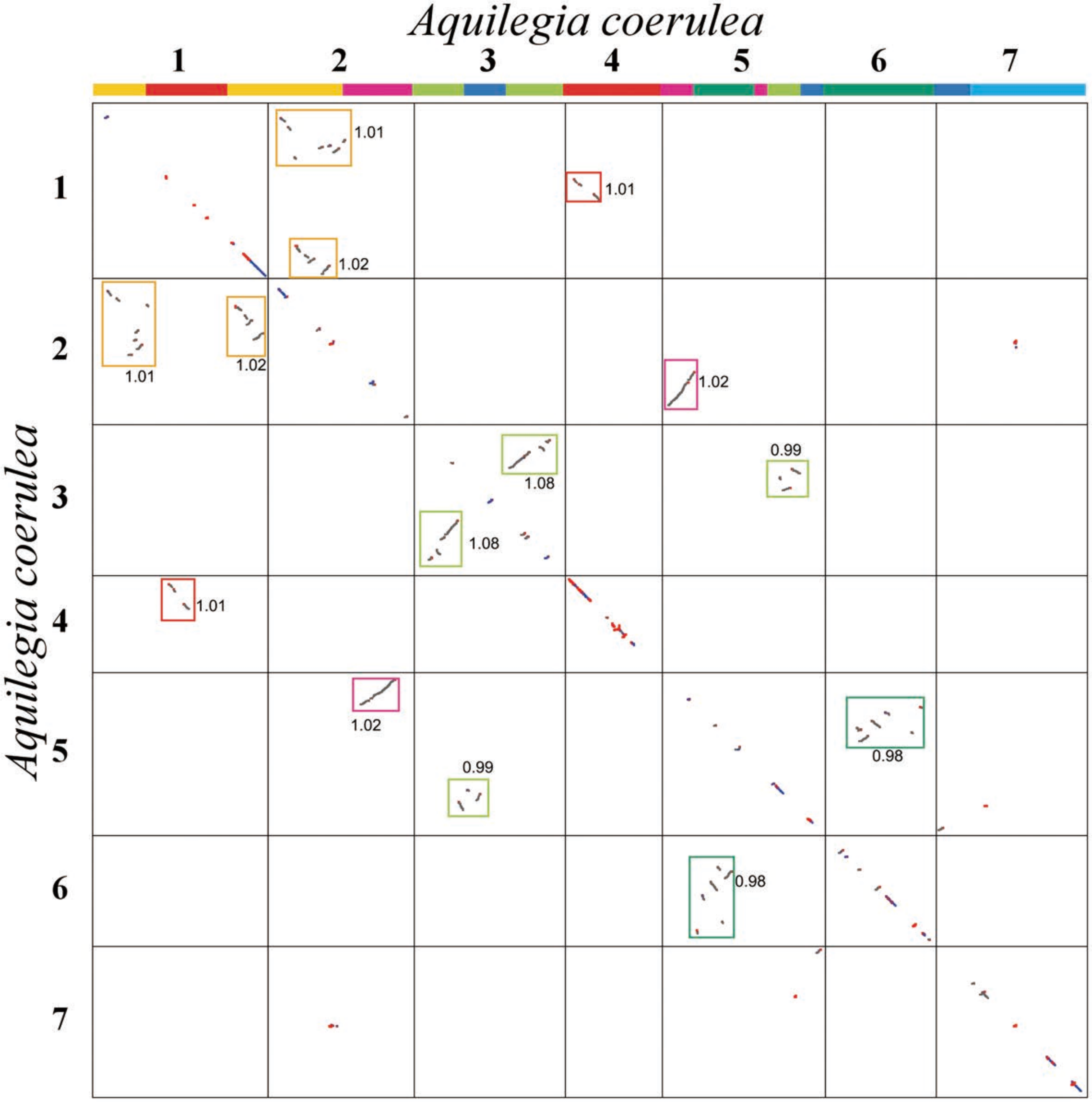

Supplementary Figure 7. Intragenomic homologous structure comparison analyses within the columbine genomes. The best, secondary, and other matched homologous gene pairs output by Blast are plotted in red, blue, and gray, respectively. The dotplot shows only relatively large paralogous regions (containing more than 8 gene pairs) in the columbine genome. The 7 columbine chromosomes are colored by the 7 eudicot ancestral chromosomes (Jaillon, et al. 2007). Paralogous regions within the genome are framed by solid line boxes of corresponding colors and the *Ks* medians of the regions are specified near the homologous gene regions.

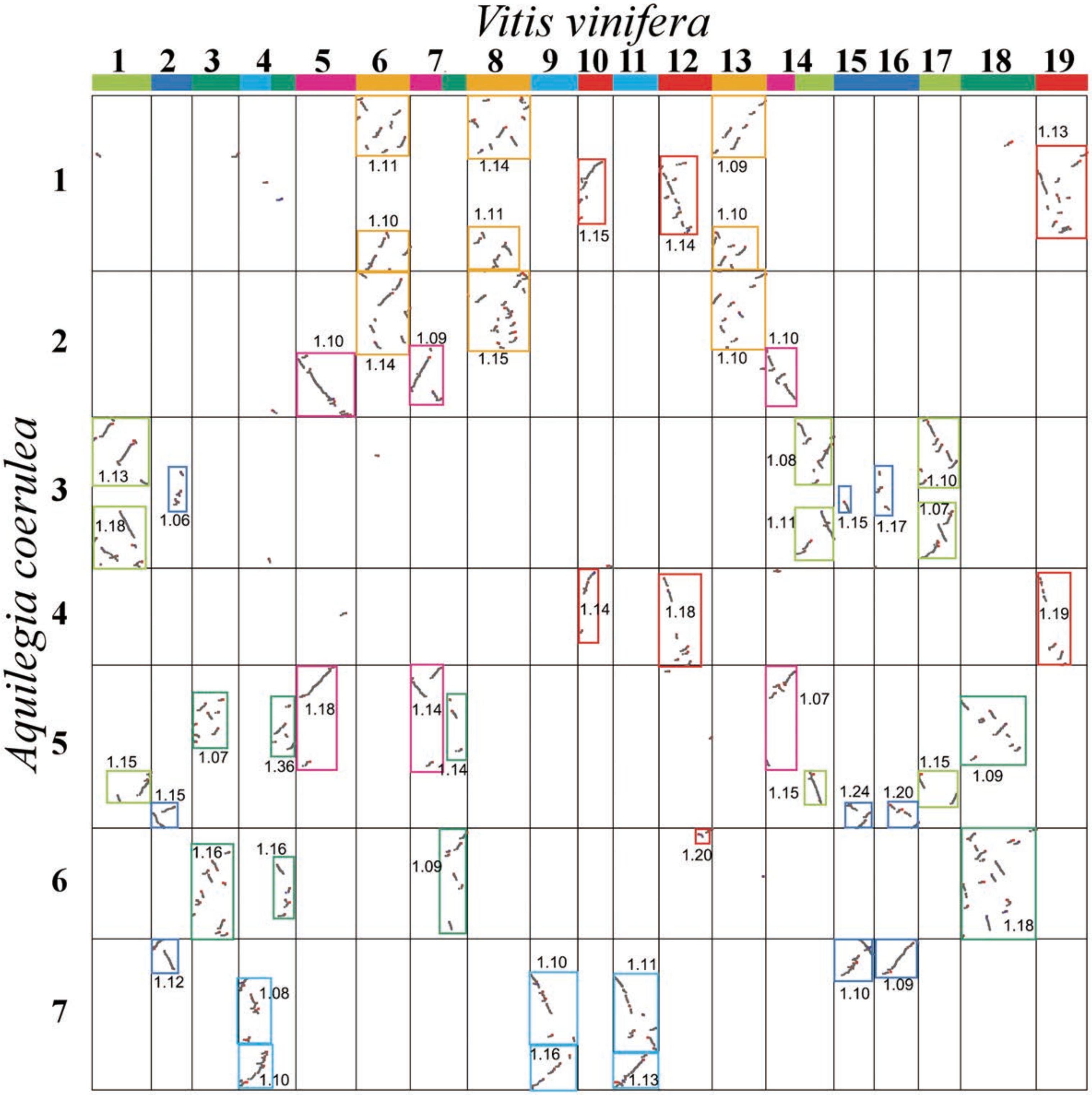

Supplementary Figure 8. Intergenomic homologous structure comparison analyses between the grape and columbine genomes. The best, secondary, and other matched homologous gene pairs output by Blast are plotted in red, blue, and gray, respectively. The dotplot shows only relatively large orthologous regions (containing more than 8 gene pairs) between grape and columbine genomes. The 19 grape chromosomes are colored by the 7 eudicot ancestral chromosomes (Jaillon, et al. 2007). Orthologous regions between the genomes are framed by solid line boxes of corresponding colors and the *Ks* medians of the regions are specified near the homologous gene regions.

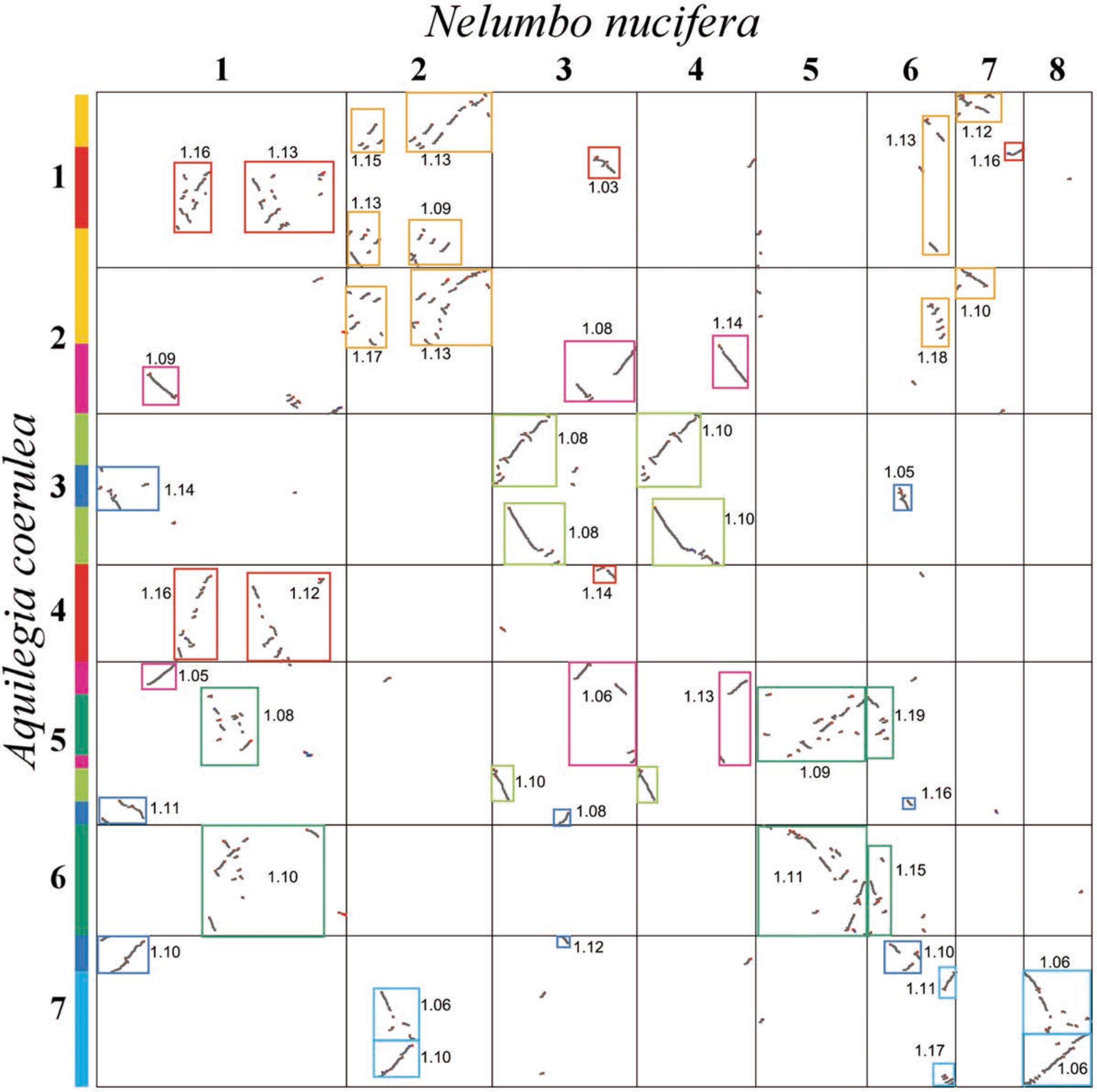

Supplementary Figure 9. Intergenomic homologous structure comparison analyses between the columbine and lotus genomes. The best, secondary, and other matched homologous gene pairs output by Blast are plotted in red, blue, and gray, respectively. The dotplot only shows relatively large orthologous regions (containing more than 8 gene pairs) between lotus and columbine genomes. The 7 columbine chromosomes are colored by the 7 eudicot ancestral chromosomes (Jaillon, et al. 2007). Orthologous regions between the genomes are framed by solid line boxes of corresponding colors and the *Ks* medians of the regions are specified near the homologous gene regions.

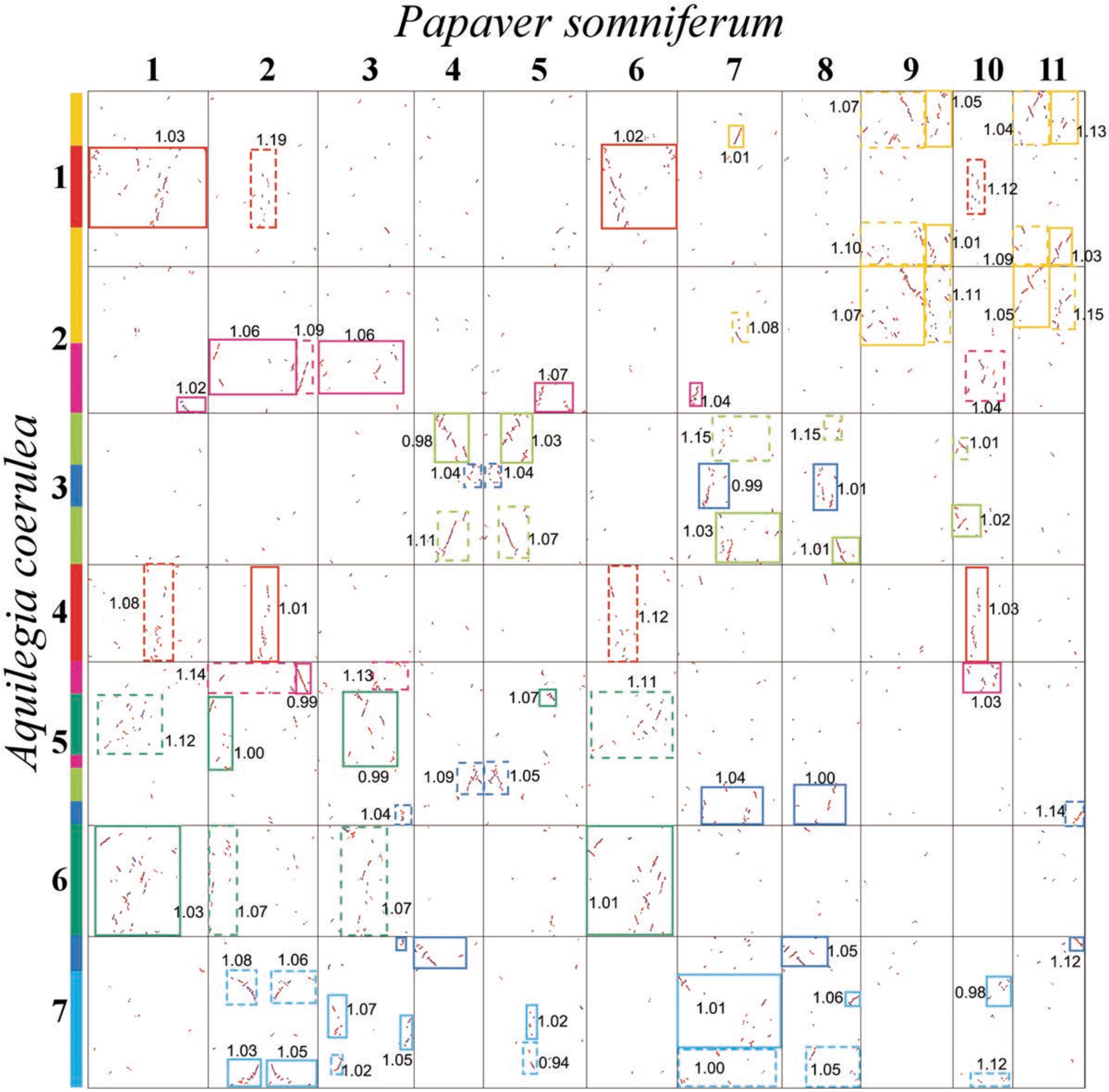

Supplementary Figure 10. Intergenomic homologous structure comparison analyses between the columbine and poppy genomes. The best, secondary, and other matched homologous gene pairs output by Blast are plotted in red, blue, and gray, respectively. The dotplot only shows relatively large orthologous regions (containing more than 6 gene pairs) between poppy and columbine genomes. The 7 columbine chromosomes are colored by the 7 eudicot ancestral chromosomes (Jaillon et al., 2007). Orthologous and paralogous regions between the genomes are framed by solid and dashed line boxes of corresponding colors, respectively and the *Ks* medians of the regions are specified near the homologous gene regions.

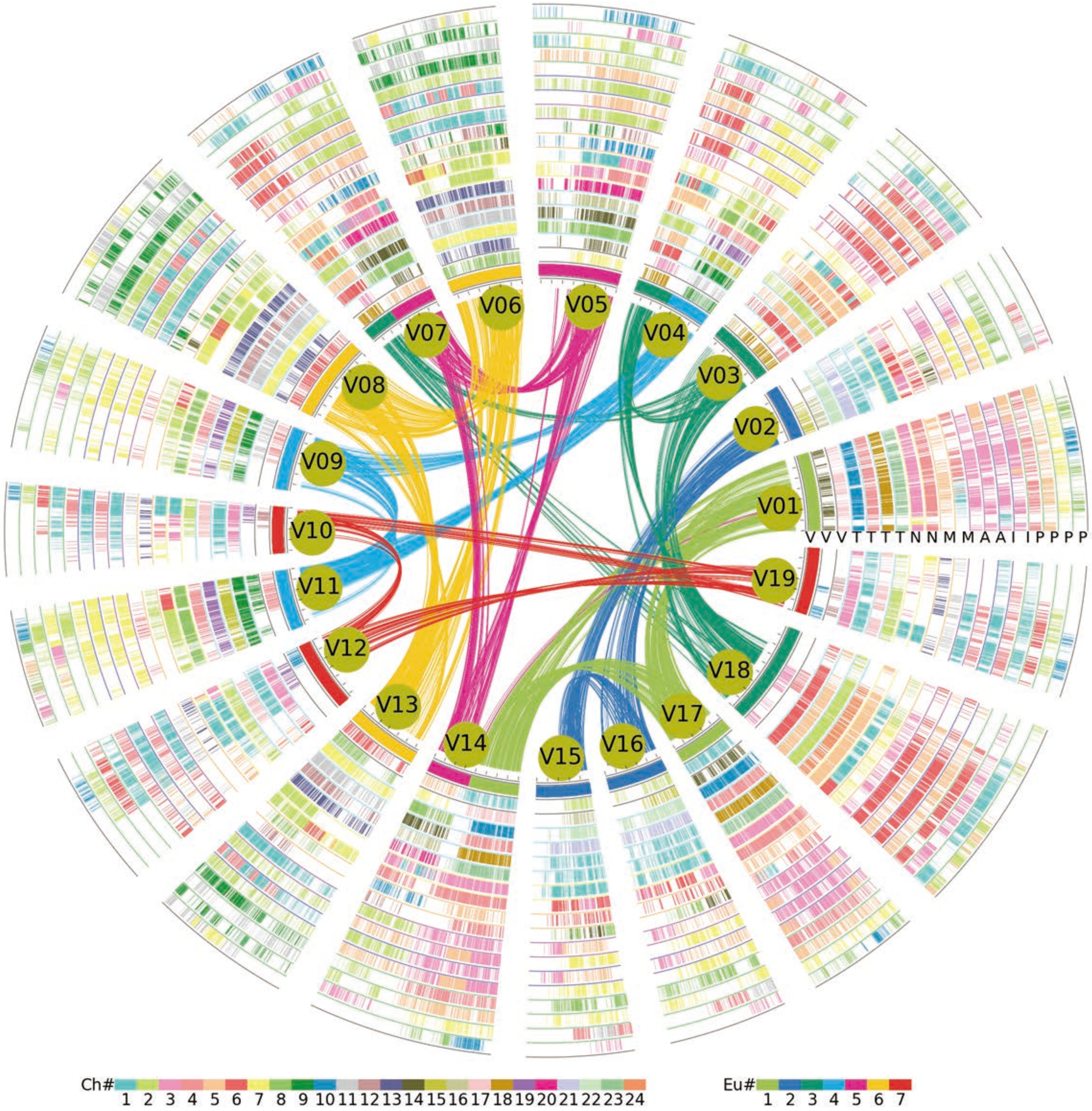

Supplementary Figure 11. Homologous alignments of selected genomes with grape as the reference. Genomic paralogy, orthology, and outparalogy information within and among species, with the same name abbreviations as per fig. 2G, are displayed in nineteen circles, each corresponding to an extant gene in fig. 2H. Eu color coding corresponds to the seven ancestral chromosomes before the ECH (Jaillon, et al. 2007), and Ch color coding represents the chromosome number of their respective source genome, as shown in the color scheme at the bottom. The short lines forming the innermost chromosome circle represent predicted genes from the reference genome, and short vertical lines forming other circles indicate homologous genes. Predicted genes in the innermost circle from the grape genome have two sets of paralogous regions, forming another two circles. Each of the three sets of grape paralogous chromosomal regions has two orthologous copies in lotus, macadamia nut, Rocky Mountain columbine and Gansu columbine var genomes respectively, and four in poppy and tetracentron genomes respectively. Finally, nineteen circles are formed with grape as reference.

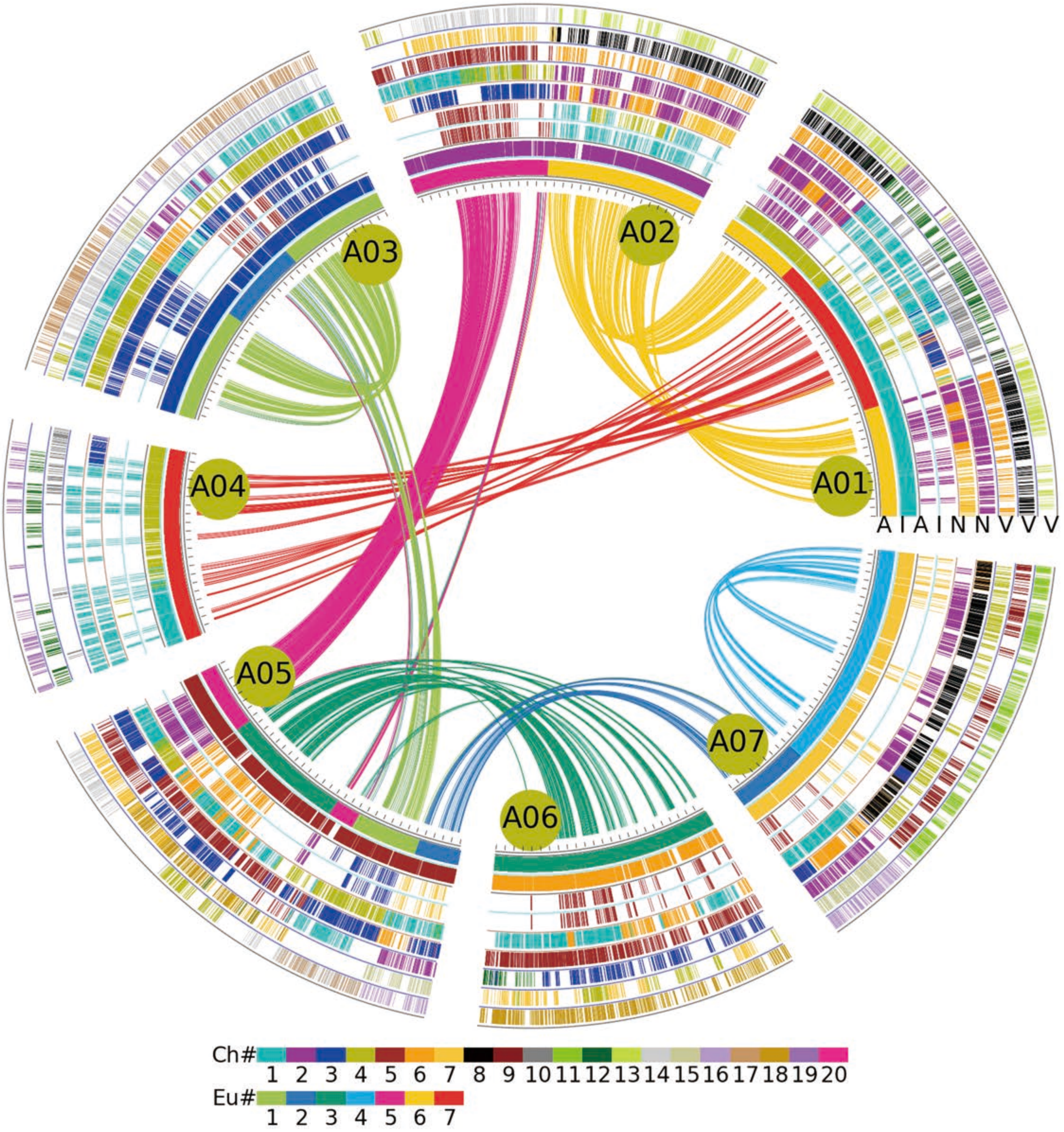

Supplementary Figure 12. Homologous alignments of selected genomes with columbine as the reference. Genomic paralogy, orthology, and outparalogy information within and among species, with the same name abbreviations as per fig. 2G, are displayed in nine circles, each corresponding to an extant gene in fig. 2G. Eu color coding corresponds to the seven ancestral chromosomes before the ECH (Jaillon, et al. 2007), and Ch color coding represents the chromosome number of their respective source genome, as shown in the color scheme at the bottom. The short lines forming the innermost chromosome circle represent predicted genes from the reference genome, and short vertical lines forming other circles indicate homologous genes. Predicted genes in the innermost circle from the columbine genome have a set of paralogous regions, forming another circle, and have two sets of paralogous regions in another columbine variety, forming two circles. Each of the two sets of columbine paralogous chromosomal regions has two orthologous copies in the lotus genome and three orthologous copies in the grape genome. Finally, nine circles are formed with columbine as the reference.

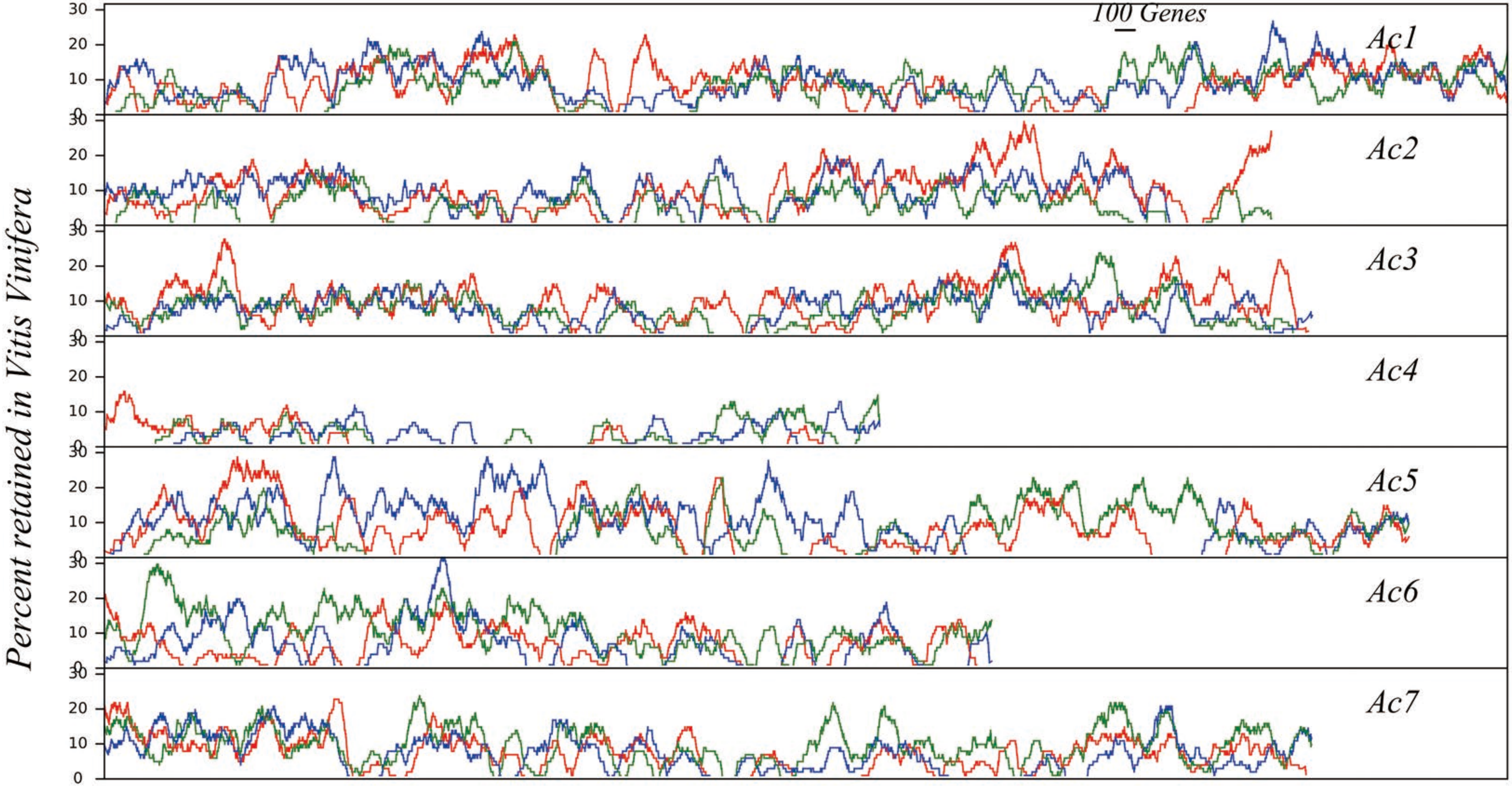

Supplementary Figure 13. Grape gene retention along the corresponding orthologous columbine chromosome. Using the columbine chromosomes as the reference, with 100 genes as a sliding window, the percentage of gene retention in the three grape subgenomes is shown in red, blue, and green.

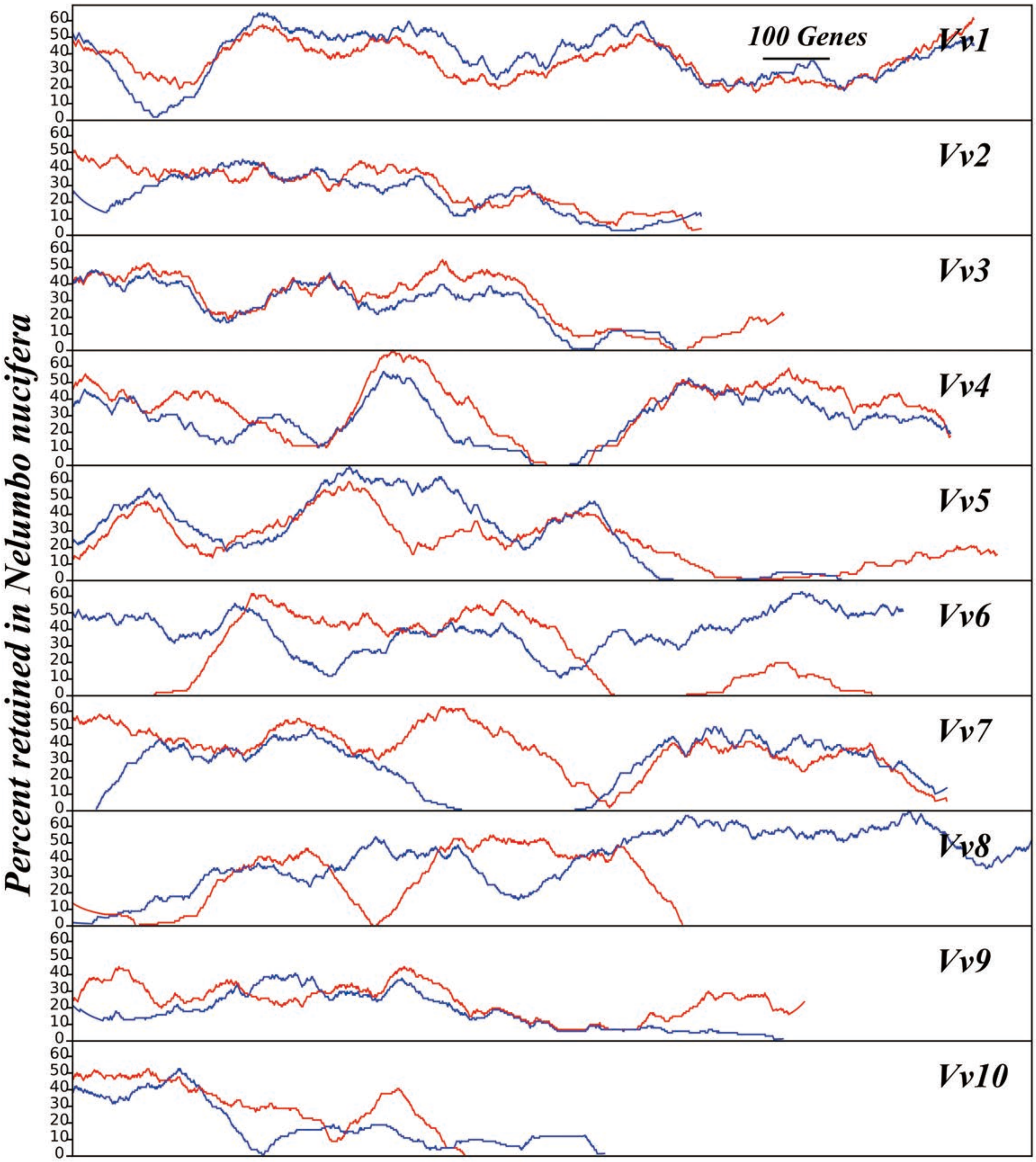

Supplementary Figure 14A. Lotus gene retention along the corresponding orthologous grape chromosome 1-10. Using the grape 1-10 chromosomes as the reference, with 100 genes as a sliding window, the percentage of gene retention in both lotus subgenomes is shown in red and blue.

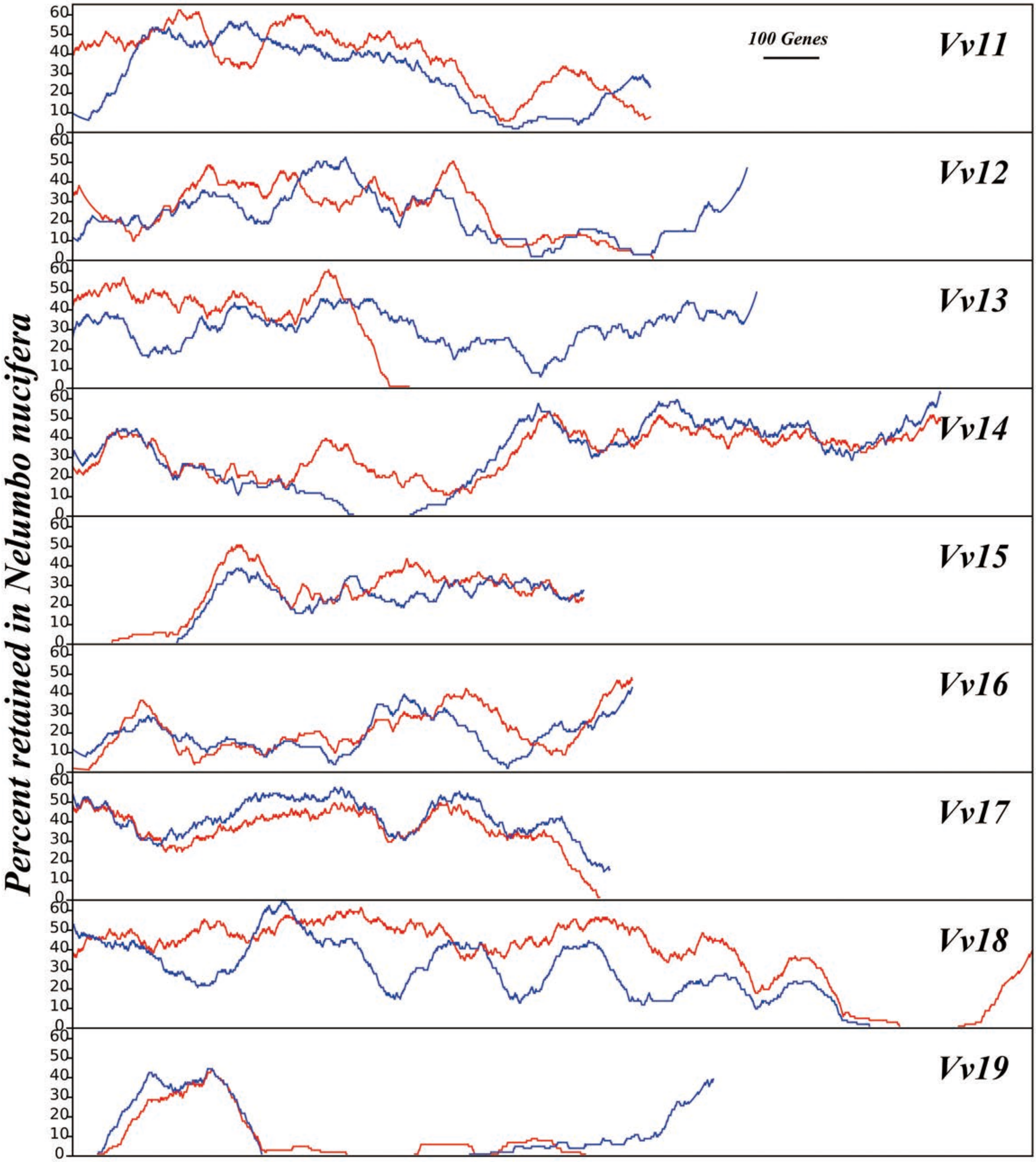

Supplementary Figure 14B. Lotus gene retention along the corresponding orthologous grape chromosome 11-19. Using the grape 11-19 chromosomes as the reference, with 100 genes as a sliding window, the percentage of gene retention in both lotus subgenomes is shown in red and blue.

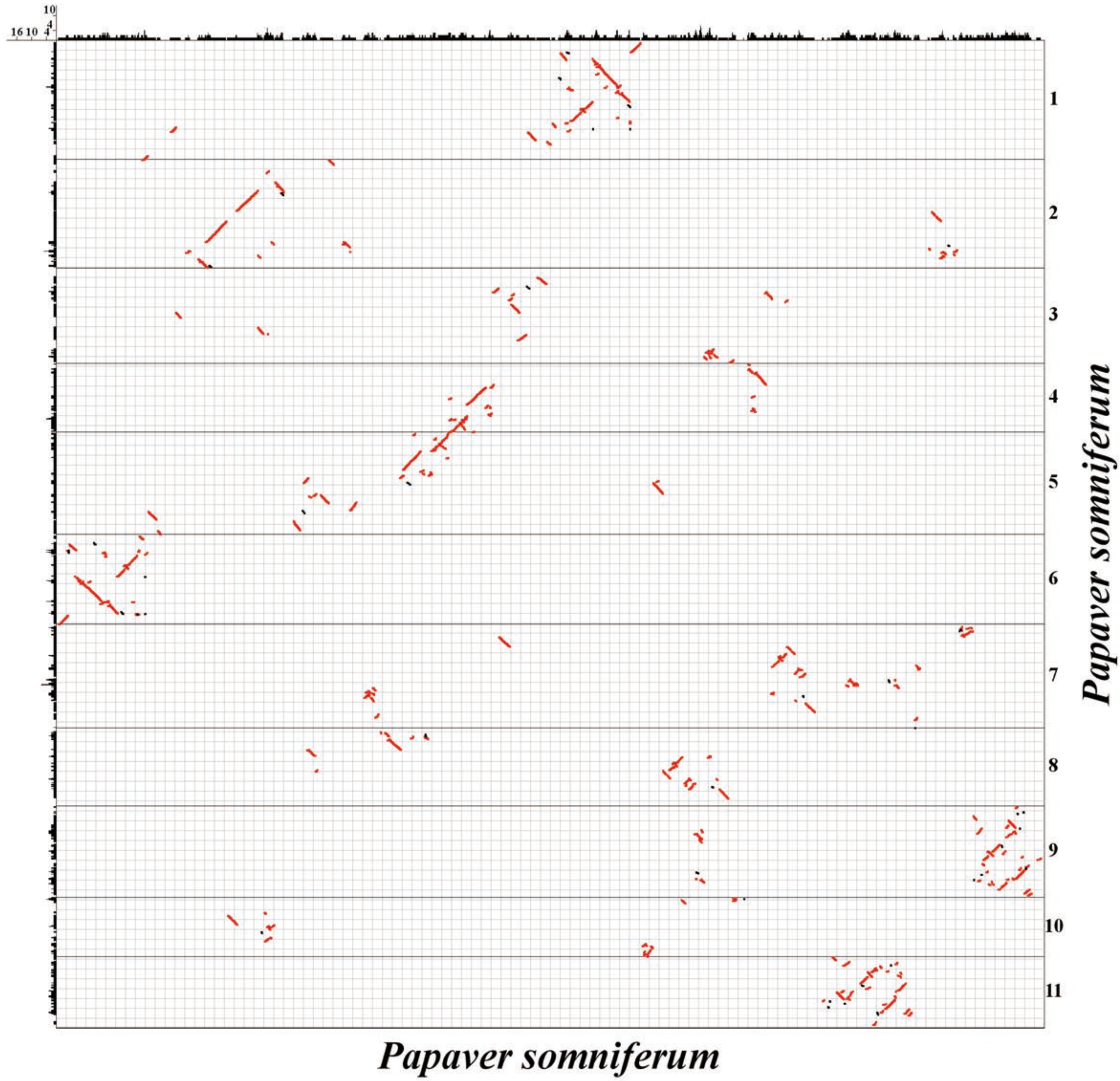

Figure S15. Homologous gene dot plot of poppy with syntenic blocks generated by Poppy Recent Teraploidy (PRT). The syntenic blocks whose *Ks* median is between 0 and 0.3 are displayed and mapped to the X and Y axis. The bigger colinear depth ratio of colinear regions is, the higher it maps to the axis.

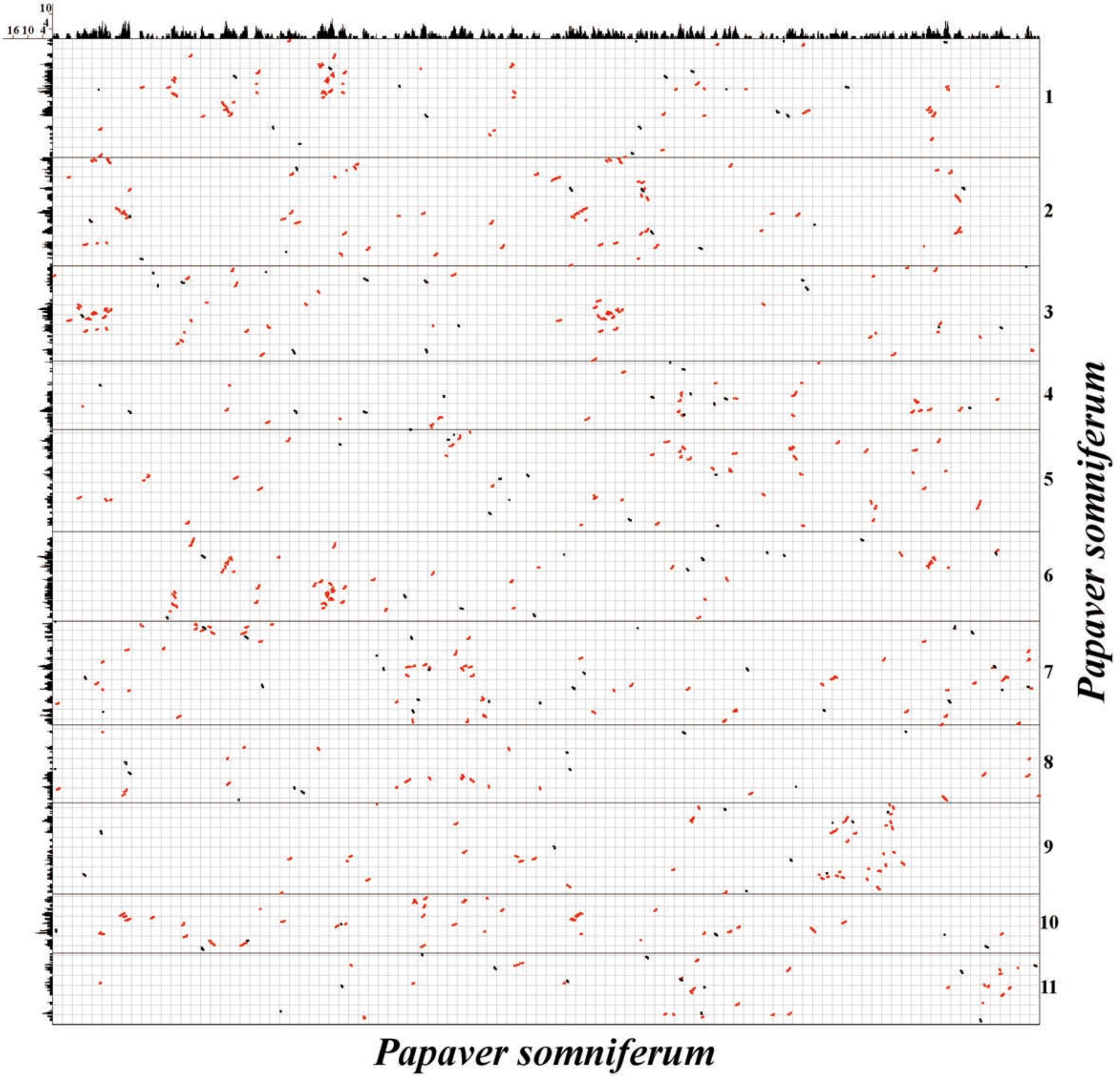

Supplementary Figure 16. Homologous gene dot plot of *P. somniferum* with syntenic blocks generated by RCT. The syntenic blocks whose *Ks* median is between 0.4 and 1.8 are displayed and mapped to the X and Y axis. The bigger colinear depth ratio of colinear regions is, the higher it maps to the axis.
