# Supplementary material for: Paleopolyploidies and Genomic Fractionation in Major Eudicot Clades
Source: Front Plant Sci. 2022 May 31;13:883140. doi: 10.3389/fpls.2022.883140 (PMC9194900; doi:10.3389/fpls.2022.883140)
Supplement: Supplementary file 2 [file Data_Sheet_2.docx]

**Supplementary text**

**Supplementary Disscusion**

**Two rounds whole-genome duplication of *P. somniferum***

The *Ks* distribution curve of *P. somniferum* displayed obvious bimodal distribution, and the two peaks of *Ks* were located at ~0.079 (±0.078) and ~1.073 (±0.232), respectively **(Figure 1A and Supplementary Table 3)**. According to the *Ks* medians of the colinear gene pairs from blocks in the *P. somniferum* genome, we could divide blocks into RCT- and PST-produced blocks. Those PST-produced blocks with *Ks* median is between 0 and 0.3, and the RCT-produced blocks with *Ks* median is between 0.4 and 1.8. The PST-produced blocks covered 82.39% (34,414/41,770 genes) of the genome **(Supplementary Figure 15 and Supplementary Table 20)** and the RCT-produced blocks covered 60.69% (25,350/41,770 genes) **(Supplementary Figure 16 and Supplementary Table 21)**. These results are very similar to the two identified WGDs (ξ and η events) produced blocks of *Selaginella* reported in previous studies, covering 64.8% and 76.7% of the genome, respectively ([Wang et al., 2020](#_ENREF_4)). Therefore, we inferred that the two rounds duplication of *P. somniferum* are two whole-genome duplication events, rather than one WGD and one segmental duplication.

**Supplementary** **materials and methods**

**Kernel function analysis of *Ks***

Distributions of synonymous nucleotide substitutions on synonymous sites (*Ks*) of homologous genes from different genomes can reflect the timing of divergence and speciations. We used the kernel function to analyze the *Ks* distribution of colinear homologs within and between genomes. The *Ks* distribution is thought of as a mix of normal distributions. The width of the kernel smoothing density function is set at 0.05 using Matlab to estimate the density of each *Ks* list and obtain the density distribution curves. The Gaussian curve was fitted using the cftool toolbox. R-squared is used to evaluate the goodness-of-fit and is generally set to at least 95%. The smallest number of normal distributions was used to represent the complex *Ks* distribution and the corresponding evolutionary event is represented by principle one. We used the maximum likelihood estimate *μ* (*Ks* peak) from *Ks* distribution curves.

**Evolutionary rate correction**

Genes in different genomes may evolve at different rates. The divergence levels between colinear homologs in different genomes or within a genome can be inferred by estimating *Ks* between colinear genes. To assess *Ks* distributions associated with polyploidization and speciation events, we used a pre-existing comprehensive correction approach ([Wang et al., 2018](#_ENREF_2); [Wang et al., 2017](#_ENREF_3); [Wang et al., 2019](#_ENREF_5)). To correct the evolutionary rates of RCT-produced duplicated genes, the maximum likelihood estimates $\mu$ from inferred *Ks* means of RCT-produced duplicated genes were aligned to the same value of that of *A. coerulea*, which has evolved to be the slowest. Supposing the *Ks* value of *A. coerulea* duplicated gene pairs is a random variable $X_{C}\sim\left(\mu_{C}，\sigma_{C}^{2}\right)$, and for a duplicated gene pair in *P. somniferum* genome, the *Ks* to be $X_{P}\sim\left(\mu_{P}，\sigma_{P}^{2}\right)$, we obtained the relative difference:$r=(\mu_{P}-\mu_{C})/\mu_{C}$.

To get the corrected $X_{P-correction}\sim\left(\mu_{P-corrrction}，\sigma^{2}_{P-correction}\right)$, we defined the correction coefficient as $\frac{\mu_{P-correction}}{\mu_{P}}=\frac{\mu_{C}}{\mu_{P}}=\lambda_{P}$,

and $\mu_{P-correction}=\frac{\mu_{C}}{\mu_{P}}\times\mu_{P}=\frac{1}{1+r}\times\mu_{P}$.

Therefore $\lambda_{P}=\frac{1}{1+r}$,

then, $X_{P-correction}\sim\left(\lambda_{P}\mu_{P}，\lambda_{P}^{2}\sigma_{P}^{2}\right)$.

To calculate *Ks* of homologous gene pairs between two plants *(i, j*), suppose the *Ks* distribution is $X_{ij}\sim\left(\mu_{ij}，\sigma_{ij}^{2}\right)$, we adopted the algebraic mean of the correction coefficients from two plants, $\lambda_{ij}=(\lambda_{i}+\lambda_{j})/2$,

then, $X_{ij-correction}\sim\left(\lambda_{ij}\mu_{ij}，\lambda_{ij}^{2}\sigma_{ij}^{2}\right)$.

Therefore, the *Ks* value between *A. coerulea* and *P. somniferum* is $X_{CP-correction}\sim\left(\lambda_{CP}\mu_{CP}，\lambda_{CP}^{2}\sigma_{CP}^{2}\right)$.

Based on *A. coerulea* simultaneously separating with *V. vinifera* and *N. nucifera*, we used the unweighted pair-group method with arithmetic means (UPGMA),

$d(A,B)=\frac{1}{\left|A\right|\left|B\right|}\sum_{x\in A} \sum_{y\in B} d(x,y)$.

Then we derived the inferred *Ks* means from *A. coerulea*-*V. vinifera* and *A. coerulea*-*N. nucifera*: $\mu_{CG-correction}=\mu_{CL-correction}=(\mu_{CG}+\mu_{CG})/2$.

The correction coefficients from *A. coerulea*-*V. vinifera* and *A. coerulea*-*N. nucifera* are $\lambda_{CG}=\mu_{CG-correction}/\mu_{CG}$, $\lambda_{CL}=\mu_{CL-correction}/\mu_{CL}$.

Therefore, the *A. coerulea*-*V. vinifera* and *A. coerulea*-*N. nucifera* corrected distributions are $X_{CG-correction}\sim\left(\lambda_{CG}\mu_{CG}，\lambda_{CG}^{2}\sigma_{CG}^{2}\right)$, $X_{CL-correction}\sim\left(\lambda_{CL}\mu_{CL}，\lambda_{CL}^{2}\sigma_{CL}^{2}\right)$.

Aligning the *V. vinifera* and *A. coerulea*-*V. vinifera* corrected distributions, we see that $\left(\frac{1+\lambda_{G}}{2}\right)\times\mu_{CG}=\mu_{CG-correction}$.

Therefore, we obtain the correction coefficient of *V. vinifera*: $\lambda_{G}=\lambda_{CG}\times2-1$.

Similarly, we achieve the correction coefficient of *N. nucifera*: $\lambda_{L}=\lambda_{CL}\times2-1$.

Then we have $X_{G-correction}\sim\left(\lambda_{G}\mu_{G}，\lambda_{G}^{2}\sigma_{G}^{2}\right)$, $X_{L-correction}\sim\left(\lambda_{L}\mu_{L}，\lambda_{L}^{2}\sigma_{L}^{2}\right)$.

Similar to the above correction, we obtain $\lambda_{GL}=(\lambda_{G}+\lambda_{L})/2$.

Finally, we see that $X_{GL-correction}\sim\left(\lambda_{GL}\mu_{GL}，\lambda_{GL}^{2}\sigma_{GL}^{2}\right)$.

**Supplementary Tables**

**Supplementary Table 1.** Number of homologous blocks and gene pairs within a genome or between genomes.

| **Homologous Blocks within and among genome** | **BL^a^ > 4** | **BL >10** | **BL >20** | **BL >50** | **ACGP^b^** | **LDB^c^** | **LDB on chromosomes** |
| --- | --- | --- | --- | --- | --- | --- | --- |
| *Vitis vinifera* | 2,330/232 | 1,470/65 | 1,079/37 | -/- | 10.04, 22.62, 29.16, - | 46 | Vvi1-Vvi14 |
| *Nelumbo nucifera* | 6,471/392 | 4,767/54 | 4,622/43 | 3,915/22 | 16.51, 88.28, 107.49, 177.95 | 618 | Nnu3-Nnu4 |
| *Aquilegia coerulea* | 1,657/215 | 673/30 | 423/11 | 207/3 | 7.71, 22.43, 38.45, 69.00 | 105 | Aco2-Aco5 |
| *Aquilegia oxysepala* | 2,670/374 | 963/34 | 737/17 | 349/4 | 7.14, 28.32, 43.35, 87.25 | 138 | Aox2-Aox5 |
| *V. vinifera vs N. nucifera* | 19,115/1,352 | 13,838/320 | 12,233/202 | 8,970/97 | 14.14, 43.24, 60.56, 92.47 | 348 | Vvi11- Nnu8 |
| *V. vinifera vs A. coerulea* | 12,165/994 | 8,517/334 | 5,886/138 | 2,626/34 | 12.24, 25.50, 42.65, 77.24 | 183 | Vvi5-Aco2 |
| *N. nucifera vs A. coerulea* | 15,678/1,071 | 11,726/326 | 9,618/167 | 6,430/62 | 14.64, 35.97, 57.59, 103.71 | 390 | Nnu4-Aco3 |

^a^BL: block_length; ^b^ACGP: average colinear gene pairs respectively per block; ^c^LDB: number of colinear gene pairs reside in longest duplicated block

**Supplementary Table 2.** Number of homologous genes residing in inferred colinear gene blocks within a genome or between genomes.

| **Homologous Blocks within and among genome** | **BL^a^ > 4** | **BL >10** | **BL >20** | **BL >50** | **LDB^b^** | **LDB on chromosomes** |
| --- | --- | --- | --- | --- | --- | --- |
| *Vitis vinifera* | 3,698 | 2,528 | 1,923 | - | 46 | Vvi1-Vvi14 |
| *Nelumbo nucifera* | 9,546 | 8,266 | 8,001 | 6,698 | 618 | Nnu3-Nnu4 |
| *Aquilegia coerulea* | 2,644 | 1,175 | 712 | 319 | 105 | Aco2-Aco5 |
| *Aquilegia oxysepala* | 4,011 | 1,661 | 1,288 | 562 | 138 | Aox2-Aox5 |
| *V. vinifera vs N. nucifera* | 11,027 vs 13,026 | 9,508 vs 11,152 | 8,577 vs 9,979 | 6,499 vs 7,538 | 348 | Vvi11- Nnu8 |
| *V. vinifera vs A. coerulea* | 9,290 vs 8,876 | 7,385 vs 6,875 | 5,300 vs 4,868 | 2,440 vs 2,355 | 183 | Vvi5-Aco2 |
| *N. nucifera vs A. coerulea* | 12,153 vs 10,042 | 10,266 vs 8,246 | 8,627 vs 6,902 | 5,982 vs 4,687 | 390 | Nnu4-Aco3 |

^a^BL: block_length; ^b^LDB: number of colinear gene pairs reside in longest duplicated block

**Supplementary Table 3.** Kernel function analysis of *Ks* distribution related to duplication events within each genome and between selected genomes (before evolutionary rate correction).

| **Intragenomic/Intergenomic colinear gene pairs** | **Weight coefficient related to duplication event or speciation** | **Peak of *Ks* distribution (μ)** | **Deviation (σ)** |
| --- | --- | --- | --- |
| ***V. vinifera* ECH-related** | 0.883 | 1.029 | 0.244 |
| ***A. coerulea* RCT-related** | 0.608 | 1.030 | 0.155 |
| ***N. nucifera* NST-related** | 0.544 | 0.492 | 0.064 |
| ***M. integrifolia* MST-related** | 0.894 | 0.415 | 0.213 |
| ***T. sinense* TAT-related** | 0.838 | 0.470 | 0.064 |
| ***T. sinense* TRT-related** | 0.957 | 0.339 | 0.058 |
| ***P. somniferum* RCT-related** | 0.646 | 1.073 | 0.232 |
| ***P. somniferum* PST-related** | 0.314 | 0.079 | 0.078 |
| ***V. vinifera*-*A. coerulea*** | 0.916 | 1.087 | 0.128 |
| ***V. vinifera*-*N. nucifera*** | 0.792 | 0.934 | 0.104 |
| ***A. coerulea*-*N. nucifera*** | 0.881 | 1.056 | 0.126 |
| ***A. coerulea*-*P. somniferum*** | 0.919 | 1.030 | 0.114 |
| ***N. nucifera*-*M. integrifolia*** | 0.917 | 0.763 | 0.189 |
| ***V. vinifera*-*T. sinense*** | 0.850 | 0.744 | 0.087 |

**Supplementary Table 4.** Kernel function analysis of *Ks* distribution related to duplication events within each genome and between selected genomes (after evolutionary rate correction).

| **Intragenomic/Intergenomic colinear gene pairs** | **Weight coefficient related to duplication event or speciation** | **Peak of Ks distribution (μ)** | **Deviation (σ)** |
| --- | --- | --- | --- |
| ***V. vinifera* ECH-related** | 0.884 | 1.000 | 0.238 |
| ***A. coerulea* RCT -related** | 0.608 | 1.030 | 0.155 |
| ***N. nucifera* NST -related** | 0.543 | 0.506 | 0.065 |
| ***P. somniferum* RCT-related** | 0.646 | 1.030 | 0.224 |
| ***P. somniferum* PST-related** | 0.316 | 0.076 | 0.077 |
| ***V. vinifera*-*A. coerulea*** | 0.917 | 1.072 | 0.127 |
| ***V. vinifera*-*N. nucifera*** | 0.793 | 0.934 | 0.104 |
| ***A. coerulea*-*N. nucifera*** | 0.881 | 1.072 | 0.127 |
| ***A. coerulea*-*P. somniferum*** | 0.918 | 1.010 | 0.113 |

**Supplementary Table 13.** *Nelumbo nucifera* gene loss and gene translocation rates with *Vitis vinifera* as the reference genome.

| ***Vitis vinifera*** | | ***Nelumbo nucifera*** | | |
| --- | --- | --- | --- | --- |
| **Chr** | **Genes** | **Para 1-2** | **Diff para1,2** | **Loss in all para** |
| **1** | 1398 | 0.61, 0.57 | 0.04 | 0.47 |
| **2** | 975 | 0.66, 0.70 | 0.04 | 0.61 |
| **3** | 1103 | 0.63, 0.64 | 0.01 | 0.57 |
| **4** | 1362 | 0.53, 0.61 | 0.08 | 0.47 |
| **5** | 1434 | 0.72, 0.60 | 0.12 | 0.62 |
| **6** | 1288 | 0.56, 0.48 | 0.08 | 0.43 |
| **7** | 1356 | 0.54, 0.57 | 0.03 | 0.47 |
| **8** | 1487 | 0.58, 0.49 | 0.09 | 0.45 |
| **9** | 1135 | 0.71, 0.81 | 0.10 | 0.69 |
| **10** | 841 | 0.57, 0.71 | 0.14 | 0.59 |
| **11** | 1081 | 0.59, 0.69 | 0.10 | 0.52 |
| **12** | 1262 | 0.64, 0.68 | 0.04 | 0.60 |
| **13** | 1280 | 0.53, 0.60 | 0.07 | 0.52 |
| **14** | 1624 | 0.61, 0.60 | 0.01 | 0.50 |
| **15** | 956 | 0.68, 0.69 | 0.01 | 0.59 |
| **16** | 1047 | 0.75, 0.74 | 0.01 | 0.69 |
| **17** | 1005 | 0.58, 0.54 | 0.04 | 0.45 |
| **18** | 1795 | 0.54, 0.60 | 0.06 | 0.48 |
| **19** | 1199 | 0.73, 0.75 | 0.02 | 0.71 |

**Supplementary Table 14.** *Aquilegia coerulea* gene loss and gene translocation rates with *Vitis vinifera* as the reference genome.

| ***Vitis vinifera*** | | ***Aquilegia coerulea*** | | |
| --- | --- | --- | --- | --- |
| **Chr** | **Genes** | **Para 1-2** | **Diff para1,2** | **Loss in all para** |
| **1** | 1398 | 0.74, 0.62 | 0.12 | 0.54 |
| **2** | 975 | 0.79, 0.67 | 0.12 | 0.61 |
| **3** | 1103 | 0.64, 0.67 | 0.03 | 0.57 |
| **4** | 1362 | 0.57, 0.71 | 0.14 | 0.53 |
| **5** | 1434 | 0.66, 0.73 | 0.07 | 0.58 |
| **6** | 1288 | 0.58, 0.72 | 0.14 | 0.53 |
| **7** | 1356 | 0.68, 0.57 | 0.11 | 0.49 |
| **8** | 1487 | 0.59, 0.71 | 0.12 | 0.53 |
| **9** | 1135 | 0.60, 0.80 | 0.2 | 0.63 |
| **10** | 841 | 0.69, 0.80 | 0.11 | 0.64 |
| **11** | 1081 | 0.58, 0.74 | 0.16 | 0.56 |
| **12** | 1262 | 0.67, 0.75 | 0.08 | 0.64 |
| **13** | 1280 | 0.58, 0.81 | 0.23 | 0.60 |
| **14** | 1624 | 0.71, 0.67 | 0.04 | 0.56 |
| **15** | 956 | 0.77, 0.63 | 0.14 | 0.62 |
| **16** | 1047 | 0.83, 0.76 | 0.07 | 0.70 |
| **17** | 1005 | 0.70, 0.62 | 0.08 | 0.51 |
| **18** | 1795 | 0.62, 0.72 | 0.10 | 0.54 |
| **19** | 1199 | 0.59, 0.70 | 0.11 | 0.51 |

**Supplementary Table 15.** *Vitis vinifera* gene loss and gene translocation rates with *Aquilegia coerulea* as the reference genome.

| ***Aquilegia coerulea*** | | ***Vitis vinifera*** | | | | |
| --- | --- | --- | --- | --- | --- | --- |
| **Chr** | **Genes** | **Para 1-3** | **Diff para1,2** | **Diff para1,3** | **Diff para2,3** | **Loss in all para** |
| **1** | 7578 | 0.89, 0.88, 0.90 | 0.01 | 0.01 | 0.02 | 0.79 |
| **2** | 6304 | 0.87, 0.88, 0.91 | 0.01 | 0.04 | 0.03 | 0.78 |
| **3** | 6525 | 0.88, 0.91, 0.90 | 0.03 | 0.02 | 0.01 | 0.80 |
| **4** | 4188 | 0.97, 0.94, 0.95 | 0.03 | 0.02 | 0.01 | 0.93 |
| **5** | 7047 | 0.88, 0.85, 0.88 | 0.03 | 0 | 0.03 | 0.78 |
| **6** | 4793 | 0.90, 0.91, 0.87 | 0.01 | 0.03 | 0.04 | 0.80 |
| **7** | 6521 | 0.90, 0.90, 0.89 | 0 | 0.01 | 0.01 | 0.80 |

**Supplementary Table 16.** *Nelumbo nucifera* gene loss and gene translocation rates with *Aquilegia coerulea* as the reference genome.

| ***Aquilegia coerulea*** | | ***Nelumbo nucifera*** | | |
| --- | --- | --- | --- | --- |
| **Chr** | **Genes** | **Para 1-2** | **Diff para1,2** | **Loss in all para** |
| **1** | 7578 | 0.78, 0.80 | 0.02 | 0.74 |
| **2** | 6304 | 0.81, 0.79 | 0.02 | 0.75 |
| **3** | 6525 | 0.82, 0.81 | 0.01 | 0.76 |
| **4** | 4188 | 0.91, 0.91 | 0 | 0.89 |
| **5** | 7047 | 0.79, 0.80 | 0.01 | 0.74 |
| **6** | 4793 | 0.82, 0.80 | 0.02 | 0.76 |
| **7** | 6521 | 0.83, 0.78 | 0.05 | 0.75 |

**Supplementary Table 17.** Observed distribution of gene loss and translocation numbers fitted by using different density curves of geometric distribution.

| **Reference genome** | **Genome** | **Parameter of geometry distribution** | **Fitness (R-square)** | **P-value (F-test)** |
| --- | --- | --- | --- | --- |
| ***Vitis Vinifera*** | *Nelumbo nucifera* | 0.4210 | 0.9818 | 0.9095 |
| ***Vitis Vinifera*** | *Aquilegia coerulea* | 0.3793 | 0.9897 | 0.9042 |
| ***Aquilegia coerulea*** | *Vitis Vinifera* | 0.1686 | 0.9795 | 0.9225 |
| ***Aquilegia coerulea*** | *Nelumbo nucifera* | 0.2365 | 0.9894 | 0.9448 |

**Supplementary Table 22.** Data sources.

| **Order** | **Species name** | **Version** | **Data source** | **Journal** | **Reference** |
| --- | --- | --- | --- | --- | --- |
| **1** | *Vitis vinifera* | Genoscope.12X | JGI (https://phytozome.jgi.doe.gov/) | Nature | Jaillon et al, 2007 |
| **2** | *Nelumbo nucifera* | v1.1 | GenBank(https://www.ncbi.nlm.nih.gov/genbank/) | The Plant Journal | Gui et al, 2018 |
| **3** | *Aquilegia coerulea* | v3.1 | JGI (https://phytozome.jgi.doe.gov/) | Genome Biology | Akoz et al, 2019 |
| **4** | *Papaver somniferum* | ASM357369v1 | GenBank(https://www.ncbi.nlm.nih.gov/genbank/) | Science | Guo et al, 2018 |
| **5** | *Macadamia integrifolia* | SCU_Mint_v3 | GenBank(https://www.ncbi.nlm.nih.gov/genbank/) | G3 (Bethesda) | Nock et al, 2020 |
| **6** | *Tetracentron sinense* | ASM1514329v1 | GenBank(https://www.ncbi.nlm.nih.gov/genbank/) | Genome biology | Liu et al, 2020 |

**Supplementary Figure Legends**

**Supplementary Figure 1. Histogram of the *Ks* distribution within and among genomes.** The horizontal axis represents the *Ks* values from small to large, and the vertical axis represents the number of colinear gene pairs (**A**) within the *N. nucifera* genome, (**B**) within the *A. coerulea* genome, (**C**) within the *V. vinifera* genome, (**D**) between the *V. vinifera* and *N. nucifera* genomes, (**E**) between the *V. vinifera* and *A. coerulea* genomes, and (**F**) between the *N. nucifera* and *A. coerulea* genomes.

**Supplementary Figure 2. *Ks* distribution of colinear genes within and among genomes.** *Ks* distribution of colinear genes between (dashed curves) and within (solid curves) genomes.

**Supplementary Figure 3. Intragenomic homologous structure comparison analyses within the *N. nucifera* genome.** The dotplot only shows relatively large paralogous regions (containing more than 8 gene pairs) in the *N. nucifera* genome. The *N. nucifera* chromosomes are colored by the 7 eudicot ancestral chromosomes ([Jaillon et al., 2007](#_ENREF_1)). Paralogous regions within the genome are framed by solid line boxes of corresponding colors and the *Ks* medians are marked near the homologous gene regions.

**Supplementary Figure 4. Intergenomic homologous structure comparison analyses between the *V. vinifera* and *N. nucifera* genomes.** The dotplot only shows relatively large orthologous regions (containing more than 8 gene pairs) between the *V. vinifera* and *N. nucifera* genomes. The *V. vinifera* chromosomes are colored by the 7 eudicot ancestral chromosomes ([Jaillon et al., 2007](#_ENREF_1)). Orthologous regions between the genomes are framed by solid line boxes of corresponding colors, and the *Ks* medians are marked near the homologous gene regions.

**Supplementary Figure 5. Intergenomic homologous structure comparison analyses between the *N. nucifera* and *M. integrifolia* genomes.** The dotplot only shows relatively large orthologous regions (containing more than 6 gene pairs) between *N. nucifera* and *M. integrifolia* genomes. The *N. nucifera* chromosomes are colored by the 7 eudicot ancestral chromosomes ([Jaillon et al., 2007](#_ENREF_1)). Orthologous regions between the genomes are framed by solid line boxes of corresponding colors, and the *Ks* medians are marked near the homologous gene regions.

**Supplementary Figure 6. Intergenomic homologous structure comparison analyses between the *V. vinifera* and *T. sinense* genomes.** The dotplot only shows relatively large orthologous regions (containing more than 8 gene pairs) between *V. vinifera* and *T. sinense* genomes. The *V. vinifera* chromosomes are colored by the 7 eudicot ancestral chromosomes ([Jaillon et al., 2007](#_ENREF_1)). Orthologous regions between the genomes are framed by solid line boxes of corresponding colors, and the *Ks* medians are marked near the homologous gene regions.

**Supplementary Figure 7. Intragenomic homologous structure comparison analyses within the *A. coerulea* genome.** The dotplot only shows relatively large paralogous regions (containing more than 8 gene pairs) in the *A. coerulea* genome. The *A. coerulea* chromosomes are colored by the 7 eudicot ancestral chromosomes ([Jaillon et al., 2007](#_ENREF_1)). Paralogous regions within the genome are framed by solid line boxes of corresponding colors and the *Ks* medians are marked near the homologous gene regions.

**Supplementary Figure 8. Intergenomic homologous structure comparison analyses between the *V. vinifera* and *A. coerulea* genomes.** The dotplot only shows relatively large orthologous regions (containing more than 8 gene pairs) between *V. vinifera* and *A. coerulea* genomes. The *V. vinifera* chromosomes are colored by the 7 eudicot ancestral chromosomes ([Jaillon et al., 2007](#_ENREF_1)). Orthologous regions between the genomes are framed by solid line boxes of corresponding colors and the *Ks* medians are marked near the homologous gene regions.

**Supplementary Figure 9. Intergenomic homologous structure comparison analyses between the *A. coerulea* and *N. nucifera* genomes.** The dotplot only shows relatively large orthologous regions (containing more than 8 gene pairs) between *N. nucifera* and *A. coerulea* genomes. The *A. coerulea* chromosomes are colored by the 7 eudicot ancestral chromosomes ([Jaillon et al., 2007](#_ENREF_1)). Orthologous regions between the genomes are framed by solid line boxes of corresponding colors and the *Ks* medians are marked near the homologous gene regions.

**Supplementary Figure 10. Intergenomic homologous structure comparison analyses between the *A. coerulea* and *P. somniferum* genomes.** The dotplot only shows relatively large homologous regions (containing more than 6 gene pairs) between *P. somniferum* and *A. coerulea* genomes. The *A. coerulea* chromosomes are colored by the 7 eudicot ancestral chromosomes ([Jaillon et al., 2007](#_ENREF_1)). Orthologous and paralogous regions between the genomes are framed by solid and dashed line boxes of corresponding colors, respectively. The *Ks* medians are marked near the homologous gene regions.

**Supplementary Figure 11. Homologous alignments of selected genomes with *V. vinifera* as the reference.** The following species are selected: *V. vinifera* (V), *T. sinense* (T), *N. nucifera* (N), *M. integrifolia* (M), *A. coerulea* (A), *A. oxysepala* (I) and *P. somniferum* (P). Eu color coding corresponds to the seven ancestral chromosomes before the ECH ([Jaillon et al., 2007](#_ENREF_1)), and Ch color coding represents the chromosome number of their respective source genome. The short lines forming the innermost chromosome circle represent predicted genes from the reference genome, and short vertical lines forming other circles indicate homologous genes.

**Supplementary Figure 12. Homologous alignments of selected genomes with *A. coerulea* as the reference.** The following species are selected: *A. coerulea* (A), *A. oxysepala* (I), *N. nucifera* (N) and *V. vinifera* (V). Eu color coding corresponds to the seven ancestral chromosomes before the ECH ([Jaillon et al., 2007](#_ENREF_1)), and Ch color coding represents the chromosome number of their respective source genome. The short lines forming the innermost chromosome circle represent predicted genes from the reference genome, and short vertical lines forming other circles indicate homologous genes.

**Supplementary Figure 13. *V. vinifera* gene retention along corresponding orthologous *A. coerulea* chromosomes.** Using the *A. coerulea* chromosomes as the reference, with 100 genes as a sliding window, the percentage of gene retention in three sets of *V. vinifera* subgenomes is shown in red, blue, and green lines, respectively.

**Supplementary Figure 14A. *N. nucifera* gene retention along corresponding orthologous *V. vinifera* chromosome 1-10.** Using the *V. vinifera* 1-10 chromosomes as the reference, with 100 genes as a sliding window, the percentage of gene retention in two sets of *N. nucifera* subgenomes is shown in red and blue lines, respectively.

**Supplementary Figure 14B. *N. nucifera* gene retention along corresponding orthologous *V. vinifera* chromosome 11-19.** Using the *V. vinifera* 11-19 chromosomes as the reference, with 100 genes as a sliding window, the percentage of gene retention in two sets of *N. nucifera* subgenomes is shown in red and blue lines, respectively.

**Supplementary Figure 15. Homologous gene dot plot of *P. somniferum* with syntenic blocks generated by PST.** The syntenic blocks whose *Ks* median is between 0 and 0.3 are displayed and mapped to the X and Y axis. The bigger colinear depth ratio of colinear regions is, the higher it maps to the axis.

**Supplementary Figure 16. Homologous gene dot plot of *P. somniferum* with syntenic blocks generated by RCT.** The syntenic blocks whose *Ks* median is between 0.4 and 1.8 are displayed and mapped to the X and Y axis. The bigger colinear depth ratio of colinear regions is, the higher it maps to the axis.

**Reference**

Jaillon, O., Aury, J. M., Noel, B., Policriti, A., Clepet, C., Casagrande, A., et al. (2007). The grapevine genome sequence suggests ancestral hexaploidization in major angiosperm phyla. *Nature* 449(7161), 463-467. doi:10.1038/nature06148

Wang, J., Sun, P., Li, Y., Liu, Y., Yang, N., Yu, J., et al. (2018). An overlooked paleotetraploidization in Cucurbitaceae. *Mol. Biol. Evol.* 35(1), 16-26. doi:10.1093/molbev/msx242

Wang, J., Sun, P., Li, Y., Liu, Y., Yu, J., Ma, X., et al. (2017). Hierarchically aligning 10 legume genomes establishes a family-level genomics platform. *Plant Physiol.* 174(1), 284-300. doi:10.1104/pp.16.01981

Wang, J., Yu, J., Sun, P., Li, C., Song, X., Lei, T., et al. (2020). Paleo-polyploidization in Lycophytes. *Genomics, Proteomics Bioinf.* 18(3), 333-340. doi:10.1016/j.gpb.2020.10.002

Wang, J., Yuan, J., Yu, J., Meng, F., Sun, P., Li, Y., et al. (2019). Recursive paleohexaploidization shaped the durian genome. *Plant Physiol.* 179(1), 209-219. doi:10.1104/pp.18.00921
